# Characterisation of a LINE-1 Insertion in the *RP1* Gene by Targeted Adaptive Nanopore Sequencing in a Family with Retinitis Pigmentosa

**DOI:** 10.1155/2024/6580561

**Published:** 2024-02-09

**Authors:** Michael P. Backlund, Pauliina Repo, Harri Kangas, Kati Donner, Eeva-Marja Sankila, Julia Krootila, Maarjaliis Paavo, Kirmo Wartiovaara, Tero T. Kivelä, Joni A. Turunen

**Affiliations:** ^1^Eye Genetics Group, Folkhälsan Research Center, Biomedicum Helsinki, Helsinki, Finland; ^2^Department of Ophthalmology, University of Helsinki and Helsinki University Hospital, Helsinki, Finland; ^3^Institute for Molecular Medicine Finland (FIMM), Helsinki Institute of Life Science (HiLIFE), University of Helsinki, Helsinki, Finland; ^4^Department of Clinical Genetics, University of Helsinki and Helsinki University Hospital, Helsinki, Finland

## Abstract

Retinitis pigmentosa (RP) is a group of inherited degenerative retinal disorders affecting more than 1.5 million people worldwide. For 30-50% of individuals with RP, the genetic cause remains unresolved by current clinical diagnostic gene panels. It is likely explained by variants in novel RP-associated genes or noncoding regulatory regions, or by complex genetic alterations such as large structural variants. Recent developments in long-read sequencing techniques have opened an opportunity for efficient analysis of complex genetic variants. We analysed a Finnish family with dominantly inherited RP affecting six individuals in three generations. Two affected individuals underwent a comprehensive clinical examination in combination with a clinical diagnostic gene panel, followed by whole exome sequencing in our laboratory. They exhibited typical signs of RP, yet initial sequence analysis found no causative variants. Reanalysis of the sequencing data detected a LINE-1 (L1) retrotransposon insertion of unknown size in exon 4 of the RP1 axonemal microtubule-associated (*RP1*) gene. The large chimeric L1 insertion that segregated with the disease was further characterised using targeted adaptive nanopore sequencing of *RP1*, allowing us to identify a 5.6 kb L1 transposable element insertion in *RP1* as the cause of RP in this family with dominantly inherited RP.

## 1. Introduction

Retinitis pigmentosa (RP), the most common group of inherited retinal diseases (IRD), is characterised by varying degrees of progressive visual loss from retinal degeneration. The disease usually begins with night blindness because of rod photoreceptor dystrophy [1, 2]. The destruction of rods usually results in tunnel vision. Although the visual acuity often is initially preserved, later cone involvement causes macular degeneration and decline of central vision. Intraretinal pigment deposits (“bone spicules”), attenuation of retinal blood vessels, and waxy pallor of the optic disk are characteristic fundus findings. RP can be nonsyndromic, affecting only the eyes, or part of a syndromic disease.

The genetic spectrum of IRD is diverse: almost 300 genes are associated with IRDs (RetNet, https://sph.uth.edu/RetNet/) of which more than 80 are linked with nonsyndromic RP [2]. In addition, a single gene can result in different IRD phenotypes or have autosomal dominant, autosomal recessive, X-linked, or digenic inheritance modes. Currently, up to 50% of patients with RP remain without a genetic diagnosis [3–5]. These are likely explained by both unidentified variants and variants currently considered as variants of uncertain significance (VUS). The as yet unidentified variants are probably in unknown genes, in noncoding regions of the genes, or are genetically complex structural variants (SVs). Specifically, the recent advent of advanced genomic technologies has revealed the substantial contribution of SVs to various human diseases, including IRDs [6–9]. Such variants include deletions, duplications, inversions, and translocations, which can substantially impact gene expression, structure, and function, thereby offering an understudied avenue for understanding the genetic basis of RP.

Most known pathogenic variants in IRD-associated genes are single nucleotide polymorphisms, small indels, and copy number variants (CNV), the last of which contribute to the pathogenesis in an estimated 9% of patients [10]. While pathogenic CNVs caused by transposable genomic elements are a known disease mechanism in patients with IRD [11–16], their contribution to the unresolved cases of RP remains unknown. The approximately 6 kb long interspersed nuclear element-1 (LINE-1 or L1) is the most abundant retrotransposon of the LINE family, estimated to make up 17% of the human genome [17]. Most L1 elements are no longer functional, but the remaining intact copies can give rise to CNVs and may contribute to disease when transposed at an unfavourable genetic location [18–20]. Short-read next-generation sequencing (NGS) techniques struggle with complex genomic rearrangements that span several kilobases in length. While several tools have been developed for detection of mobile element insertions from short-read sequencing data, they are easily missed in routine clinical sequencing unless specifically looked for [21, 22]. Also, the size and sequence composition of these insertions remain often unresolved from short-read sequencing data since only reads overlapping with the insert and the target gene can usually be uniquely mapped to the reference genome. Long-read sequencing methods, like nanopore sequencing, provide a powerful tool to overcome this problem [23, 24].

To uncover difficult-to-find genetic sequence variants that may contribute to IRD, we utilised third-generation, targeted adaptive nanopore sequencing, in analysis of a Finnish family with dominantly inherited RP. As a result, we characterised the size and assembly of a novel 5.6 kb LINE-1 insertion in *RP1*.

## 2. Materials and Methods

### 2.1. Patients

A Finnish family with six individuals affected by dominantly inherited RP was invited to participate. Informed written consent was obtained from all individuals. Affected maternal cousins from the third generation, III.1 and III.2 (index), consented to DNA sampling, comprehensive ophthalmic examination, and collection of medical and family history details by the Inherited Eye Diseases Service, Department of Ophthalmology, Helsinki University Hospital, Finland. Additionally, DNA was obtained from the affected mother, II.3, and healthy father, II.4, of the index patient. The study was approved by the Ethics Review Board of the Hospital District of Helsinki and Uusimaa and adhered to the tenets of the Declaration of Helsinki.

The fundi of III.1 and III.2 were imaged in colour using Clarus 500 (Carl Zeiss, Oberkochen, Germany) and spectral-domain optical coherence tomography (SD-OCT) combined with fundus autofluorescence (FAF) imaging (488 nm excitation; Spectralis, Heidelberg Engineering, Heidelberg, Germany). The visual fields were acquired using the Goldmann perimeter. Full-field electroretinogram (ERG) was recorded using the RETI-port/scan 21 unit (Roland Consult Stasche & Finger, Brandenburg an der Havel, Germany) according to the International Society for Clinical Electrophysiology of Vision standards [25].

### 2.2. Gene Panel and Whole Exome Sequencing

Genomic DNA (individuals III.1 and III.2), extracted from the peripheral blood using standard methods, was analysed using Blueprint Genetics Retinal Dystrophy Panel Plus (version 6, Feb 22, 2020) (Blueprint Genetics, Helsinki, Finland). The subsequent whole exome sequencing (WES) was performed at the Institute for Molecular Medicine Finland (FIMM, Helsinki, Finland) as follows; 50 ng of gDNA was processed according to Twist Human Core Exome EF Multiplex Complete kit (Twist Bioscience, San Francisco, CA, USA) manual. 4 *μ*l of 15 *μ*M adapters used for ligation were unique dual index (UDI) oligos with unique molecular barcodes (UMI) by IDT (Integrated DNA Technologies, Coralville, IA, USA). Library quantification and quality check were performed using LabChip GX Touch HT High Sensitivity assay (PerkinElmer, Waltham, MA, USA) and Qubit Broad Range DNA Assay (Thermo Fisher Scientific, Waltham, MA, USA). Libraries were pooled to 8-plex reactions according to concentration. The exome enrichment was performed using Twist Comprehensive Exome probes (Twist Bioscience). The captured library pools were quantified for sequencing using the KAPA Library Quantification Kit (KAPA Biosystems, Wilmington, MA, USA) and LabChip GX Touch HT High Sensitivity assay. Sequencing was performed with the Illumina NovaSeq system using an S4 flow cell with a lane divider (Illumina, San Diego, CA, USA) and v1.5 chemistry. The read length for the paired-end run was 2 × 151 bp. UMI information was not included in the sequencing reads and not used in the following analysis. The analysis was done using the Illumina DRAGEN system (Illumina). For the analysis, Illumina DRAGEN analysis pipeline v3.9 was used to look for germline, structural, and copy number variants against the reference genome build GRCh38. The identified variants were visualised using Integrative Genomics Viewer (IGV, https://www.igv.org/).

### 2.3. Targeted Adaptive Nanopore Sequencing

Targeted adaptive nanopore sequencing, targeting inherited ocular disease genes and 15 kb of adjacent regions, was performed at FIMM. The target gene panel (Supplementary Table S1), including *RP1*, was combined from retinal disorders (v2.195), structural eye disease (v1.3), corneal abnormalities (v1.12), optic neuropathy (v2.2), and infantile nystagmus (v1.4) panels available at https://panelapp.genomicsengland.co.uk/panels/. The coordinates for the targets were extended 15 kb up- and downstream. A .bed file was created by merging any overlapping regions, and Bedtools (v2.30) [26] was used to extract the sequence of the target regions from GRCh38.p13 (GCA_000001405.28). The resulting fasta file was used as the target for enrichment in sequencing.

For sequencing, 12 *μ*g of DNA diluted to 100 ng/*μ*l in EB elution buffer (Qiagen, Hilden, Germany) was sheared to ~20 kb fragments using g-TUBE (Covaris, Woburn, MA, USA). The sheared DNA was concentrated, and <10 kb fragments were depleted using an SRE XS kit (PacBio, Menlo Park, CA, USA). Three sequencing libraries per sample were prepared using the ligation sequencing kit SQK-LSK110 according to the manufacturer's standard protocol, except using 1.5 *μ*g DNA as input. The samples were sequenced adaptively for 72 h with a MinION Mk1B sequencer controlled by MinKNOW v.22.05.5 on Flow Cell R9.4.1 to enrich the target. The flow cells were washed at 21 h (III.1), 42 h (III.2), and 48 h (III.1) using the Flow Cell Wash Kit (all from Oxford Nanopore Technologies, Oxford, UK) whereafter more of the sequencing libraries were loaded to the flow cells. The total amount of libraries loaded to the flow cells was 2772 ng (III.1) and 1844 ng (III.2).

Bases were called from the resulting fast5 files using Guppy (v6.1.7) (Oxford Nanopore Technologies) with a super accuracy model for Flow Cell R9.4.1 (config file: dna_r9.4.1_450bps_sup.cfg). Only reads > 1 kb were preserved and mapped to GRCh38.p13 reference using minimap2 (v2.24) [27]. The sequencing results were visualised using IGV. A consensus sequence was created from the inserts that were fully covered by nanopore sequencing using the MUSCLE multiple sequence alignment tool provided with Unipro UGENE (v49.0) [28]. The insertions were analysed using the Dfam database (https://www.dfam.org/) and the NCBI nucleotide BLAST tool (https://blast.ncbi.nlm.nih.gov/).

### 2.4. Validation of Breakpoints

The L1 insertion breakpoints were validated by the Sanger sequencing. PCR amplification of the breakpoints was done using primer pairs with one primer in the *RP1* gene and one in the L1 insert. The L1-annealing primers are somewhat unspecific as the L1-derived sequences are found across the human genome in both orientations [29, 30]. The PCR reactions were performed using Biotools DNA polymerase according to the manufacturer's instructions (Biotools B&M Labs, Madrid, Spain), run in 1% agarose gel, and observed for L1 insertion-specific bands. For the Sanger sequencing, bands of interest were extracted using NucleoSpin Gel and PCR Clean-up Mini Kit according to the manufacturer's instructions (Macherey-Nagel, Düren, Germany). The PCR fragment of the 5′ insertion breakpoint was labelled with BigDye Terminator v3.1 Cycle Sequencing Kit (Applied Biosystems, Thermo Fisher Scientific, Waltham, MA, USA) and sequenced at FIMM. The sequencing of the 3′ breakpoint necessitated cloning into a pCR4-TOPO TA vector using standard methods. Briefly, the cloning was done with TOPO TA Cloning Kit for Sequencing (Thermo Fisher Scientific) followed by transformation into One Shot TOP10 chemically competent *E. coli* and single clone selection according to manufacturer's instructions (Thermo Fisher Scientific). The plasmids were extracted using the NucleoSpin Plasmid Mini Kit (Macherey-Nagel) and prepared for the Sanger sequencing using BigDye Terminator v3.1 (Applied Biosystems) as described above. Universal T3 and T7 sequencing primers were provided with the TOPO TA Cloning Kit for Sequencing (Thermo Fisher Scientific). For detailed information on primers, PCR conditions, and sequencing, see Supplementary File S1.

## 3. Results

### 3.1. Phenotype of the Patients

**Figure 1:**
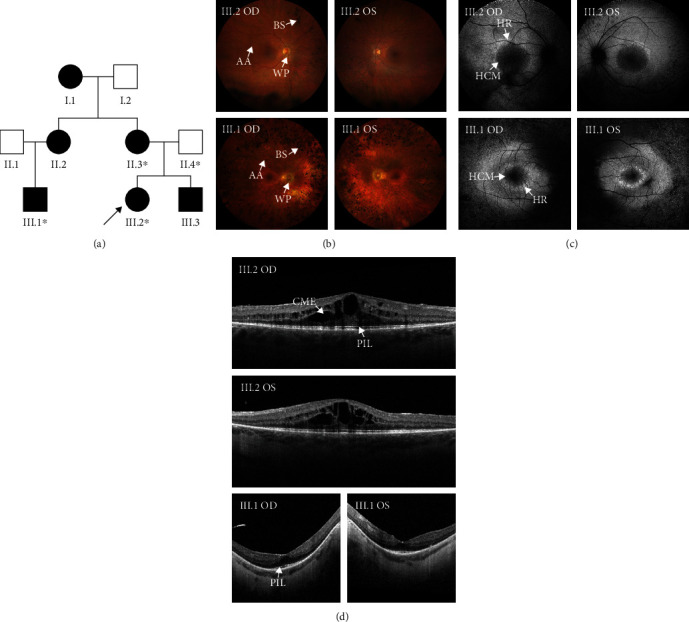
Pedigree and retinal imaging of a Finnish family with dominantly inherited retinitis pigmentosa in three generations. (a) Pedigree. Patients III.1 and III.2 (index patient, *arrow*) and an affected (II.3) and unaffected relative (II.4) participated in the study (*asterisks*). (b) Fundus images of III.1 and III.2 show arteriolar attenuation (AA), waxy optic disk pallor (WP), and bone spicule pigmentation (BS). (c) Fundus autofluorescence imaging shows a hyperautofluorescent ring (HR) around a hypoautofluorescent central macula (HCM) and in the midperiphery in both. (d) Spectral-domain optical coherence tomography shows cystoid macula oedema (CME) in III.2 and loss of the photoreceptor integrity line (PIL) in III.1. OD: oculus dexter (right eye); OS: oculus sinister (left eye).

The family with dominantly inherited RP in three generations (Figure 1(a)) reports six relatives affected by RP, two affected members from third generation, III.1 and III.2, being available for detailed examination. The inheritance pattern observed in the family is consistent with either autosomal dominant or maternal inheritance. However, since the affected members do not have syndromic RP, mitochondrial DNA is unlikely to harbour pathogenic variants [31]. The index patient III.2 was diagnosed with RP eight years ago in her thirties. Visual acuity fluctuates because of macular oedema for which she has received several intravitreal injections of triamcinolone and dexamethasone intravitreal implant with good response, each lasting 4-6 months. The Goldmann visual fields exhibit midperipheral scotomas. ERG reveals isoelectric scotopic responses and diminished photopic ones. Patient III.1, her maternal cousin, is in his sixties and was diagnosed with RP in childhood. His visual acuity is near full (best corrected, 0.8/0.8), but the Goldmann visual fields are constricted to 15-20°.

Both patients show typical RP by imaging: arteriolar attenuation, waxy pallor of the optic disk, and midperipheral bone spicules (Figure 1(b)); hypoautofluorescent central macula surrounded by a hyperautofluorescent ring in FAF (Figure 1(c)); and cystoid macular oedema in patient III.2 and perimacular loss of the photoreceptor integrity line in patient III.1 in SD-OCT (Figure 1(d)).

### 3.2. Sequencing Indicates a Transposable Element Insertion in the RP1 Gene

**Figure 2:**
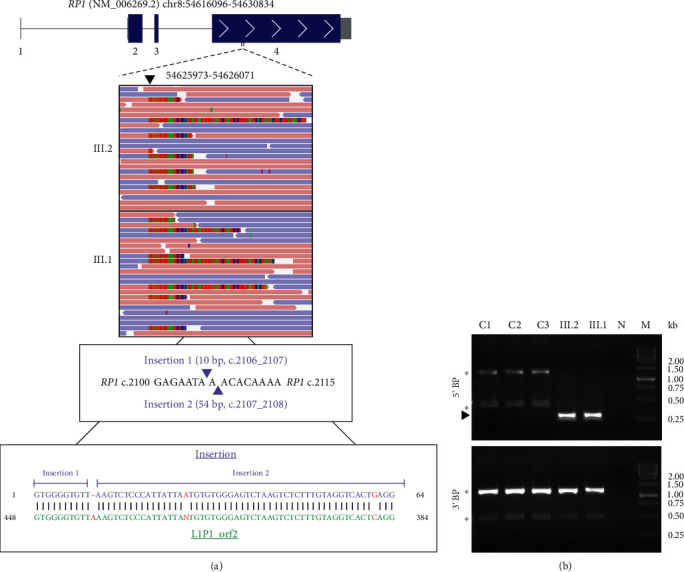
Whole exome sequencing identifies an L1 fragment insertion in *RP1* of both patients III.1 and III.2. (a) *RP1* transcript NM_006269.2 showing coding exons (blue boxes), noncoding exons (grey boxes), introns (grey lines), and exon numbers (below the exons). An Integrative Genomics Viewer (IGV) view, including soft-clipped bases of the sequencing reads, indicates a misalignment (*arrowhead*, top panel). A schematic representation of the adjacent insertion sites in *RP1* exon 4 called by DRAGEN (middle panel). The misaligned 64 bp, called by DRAGEN, aligns with the L1P1_orf2 consensus sequence from the Dfam database (DF0000316, https://www.dfam.org) (bottom panel). GRCh38 was used as the reference. (b) PCR over the predicted 5′ and 3′ breakpoints (BP) from healthy controls (C1-C3) and two affected patients (III.1, III.2). Specific PCR products of the expected size are indicated (*arrowhead*). Unspecific PCR products likely caused by L1-specific primers are indicated (*asterisk*). Lanes: M: marker; N: negative PCR control.

For both patients, the initial retinal dystrophy gene panels were negative for disease-associated IRD variants. Later reanalysis of the gene panel identified a L1 retrotransposon insertion, *RP1* c.2106_2107insL1, of unknown size in exon 4 of patient III.1. Similarly, the WES identified ambiguous insertions with varying lengths at adjacent genomic positions of exon 4 in *RP1* of III.1 that were analysed further using IGV (Figure 2(a)). Although not recognised by the WES variant calling algorithm, the variant was identified also in III.2 by IGV inspection (Figure 2(a)). Also, a requested reanalysis of the III.2 gene panel data detected the *RP1* c.2106_2107insL1. The called insertion sequence aligned with the consensus sequence of a LINE-1 subfamily, L1P1_orf2 (DF0000316, https://www.dfam.org/) in an antisense orientation (Figure 2(a)). While the 5′ breakpoint of the insertion was verified using the Sanger sequencing, the second breakpoint could not be determined, indicating that the insertion might be considerably larger than initially predicted (Figure 2(b)).

### 3.3. Targeted Adaptive Nanopore Sequencing Describes a 5.6 kb LINE-1 Insertion in RP1 Segregating with RP

**Figure 3:**
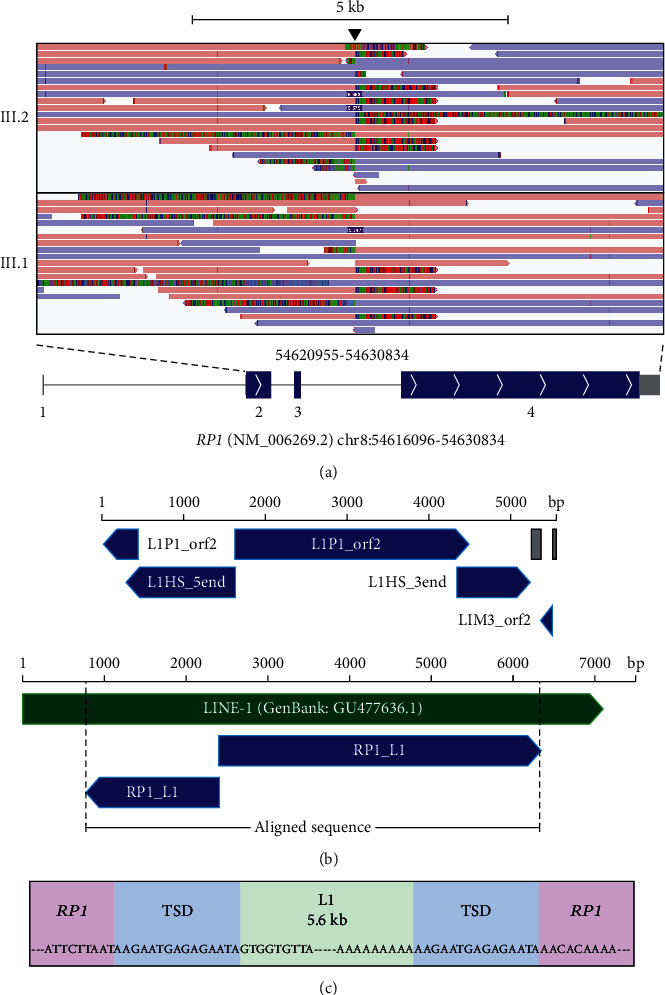
Targeted adaptive nanopore sequencing reveals the L1 insertion to be ~5.6 kb in size. (a) Integrative Genomics Viewer (IGV) view of the aligned nanopore sequencing reads over exons 2-4 of *RP1* gene (NM_006269.2). The location of the L1 insertion in exon 4 is indicated (*arrowhead*). Misaligned (soft-clipped) bases are shown to visualise sequencing reads that cover the L1 insert only partially. Misaligned bases to the left of the arrowhead show the 3′ end of the L1 insert ending with a pA track, whereas misaligned bases to the right of the arrowhead indicate the 5′ end of the L1 insert. Below is a schematic representation of transcript NM_006269.2 showing coding exons (blue boxes), noncoding exons (grey boxes), introns (grey lines), and exon numbers (below the exons). GRCh38 was used as the reference. (b) A schematic representation of the sequence structure of the L1 insertion, comprised of parts of various LINE-1 subfamily members in both orientations, according to the Dfam database (https://www.dfam.org/) (top panel). Simple repeat sequences are indicated in grey. A schematic representation of sequence alignment of the L1 insertion (RP1_L1) with a LINE-1 sequence (GenBank: GU477636.1) (bottom panel). (c) 5′ and 3′ breakpoints of the L1 insertion site (green background), showing the *RP1*-derived target site duplication (TSD) (blue background) and the *RP1* sequence around the insertion site (purple background).

To characterise the complete L1 insertion, the samples were subjected to targeted adaptive nanopore sequencing. This resulted in a total of 5.23 Gb (III.1) and 6.27 Gb (III.2) of quality control passed bases in reads of 1 kb or longer accounting for a total number of 480560 (III.1) and 579876 (III.2) reads with an average length 10.8 kb. For both samples, 19.2% of the reads were within the target panel. The average coverage of the nanopore reads for the target genes in the custom panel was 11.1 and 13.3, while for the *RP1* gene, the coverage was 13.3 and 14.0 for patients III.1 and III.2, respectively. The full L1 insert was covered by two reads in patient III.2 and in one read in patient III.1 (Figure 3(a) and Supplementary File S2).

The consensus sequence of the L1 insertion, with a size of 5571 bp, aligns with several L1 subfamily members of the Dfam database (https://www.dfam.org/) in both sense and antisense orientations (Figure 3(b) and Supplementary File S2). The L1 insertion is flanked on both sides by an *RP1*-derived 15 bp target site duplication (TSD; Figure 3(c)), and the rest of the insert aligns with 99.44% sequence identity with a previously published L1 sequence (GenBank: GU477636.1) although missing 777 bp from both ends [32]. Furthermore, the first 1635 bases of the 5′ end of the insert contain the L1 sequence in an inverted orientation, and the 3′ end harbors a 15-base longer poly-A sequence than annotated for GU477636.1. According to the HGVS guidelines, the insertion can be annotated as NG_009840.3(NM_006269.2):c.2106_2107ins[GU477636.1:g.778_2412inv;GU477636.1:g.2399_6325;A[15];2092_2106] NG_009840.3(NP_006260.1):p.(I702_N703insVGC∗). For clarity, we will refer to the insertion as *RP1* c.2106_2107insL1 in the remaining parts of the text.

**Figure 4:**
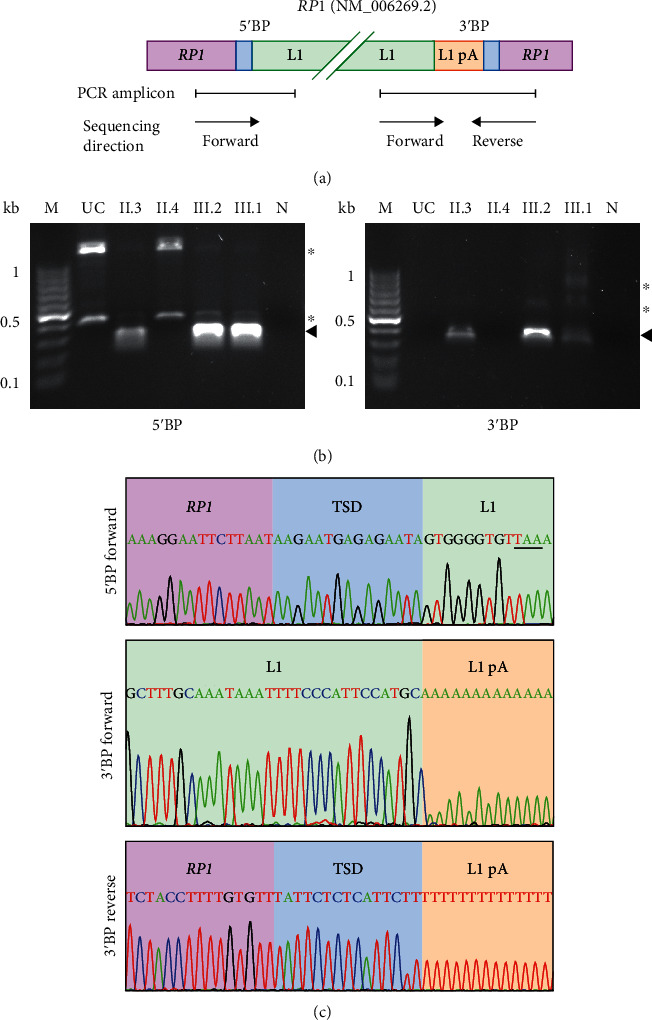
Breakpoint PCR and the Sanger sequencing confirm the L1 insertion. (a) A schematic figure of the L1 insertion (green) in exon 4 of the *RP1* gene (NM_006269.2) (purple). The pA-tail of L1 is shown in orange, and the target site duplication (TSD) is in blue. Primers were designed to amplify the 5′ and 3′ breakpoints (BP), and the PCR amplicons over the 5′ BP and 3′ BP are indicated, as well as the sequencing directions used for the Sanger sequencing. (b) PCR reactions representing the 5′ BP and 3′ BP of the L1 insertion. Specific PCR products of the expected size are indicated (*arrowhead*). Unspecific PCR products likely caused by L1-specific primers are indicated (*asterisk*). Lanes: M: marker; UC: unrelated healthy control; II.3: affected relative; II.4: unaffected relative; III.1 and III.2: patients; N: negative PCR control. Products of the expected size were amplified from the DNA samples of the affected patients (III.1, III.2) and the affected relative (II.3). No specific PCR product was amplified from the unrelated control or the unaffected relative (II.4), indicating that they lacked the L1 insertion. (c) Representative schematic figures of the insertion of BP Sanger's sequencing results. Sequencing over the 5′ BP (top panel) was done by directly sequencing the PCR product isolated from agarose gel. Because of the 3′ terminal poly-A (pA) tract of L1, the PCR product was cloned into a plasmid vector before sequencing (middle and bottom panels). The sequence preceding the pA tract aligned with the L1 subfamily L1M3_orf2 subfamily in antisense orientation and the sequence succeeding the pA tract aligned with *RP1*, including the TSD. For coding of background colouring of the sequences, see (a). The premature stop codon introduced by the L1 insertion is underlined (top panel).

The insertion segregated with RP in affected individuals. Insertion breakpoints were amplified with overlapping PCR from the DNA samples of three available affected individuals (III.1, III.2, and II.3; Figures 4(a) and 4(b)). No L1 insertion-specific amplification was observed in the healthy father of the index (II.4) nor in an unrelated healthy control (Figure 4(b)). The presence of the L1 insertion in affected individuals was confirmed by the Sanger sequencing with results consistent with the nanopore sequencing data (Figure 4(c)).

Taken together, we describe a novel 5.6 kb LINE-1 transposable element insertion in exon 4 of *RP1* in a Finnish family with dominantly inherited RP. The insertion creates a premature stop codon (underlined in Figure 4(c)) in exon 4, 10 bp downstream from the insertion site (Figure 4(c)). Wild-type *RP1* encodes a protein of 2,156 amino acids (NM_006269.2). If expressed, the L1 insertion would lead to a truncated RP1 protein of 705 amino acids where the three last ones would be encoded by the L1 insertion NG_009840.3(NP_006260.1):p.(I702_N703insVGC∗). The variant is missing from the general population according to the gnomAD database (https://gnomad.broadinstitute.org/), and loss-of-function variants in *RP1* are a known cause of dominantly inherited RP [33–36]. Following the guidelines provided by the American College of Medical Genetics, the *RP1* c.2106_2107insL1 is interpreted as pathogenic (PVS1, PM2, PP1) [37].

## 4. Discussion

In the current study, we describe a heterozygous 5.6 kb LINE-1 transposable element (TE) insertion in exon 4 of *RP1* (NM_006269.2) that segregates with the disease in a Finnish family with dominantly inherited RP. Although inherited recessively, an insertion of another TE, Alu (c.4052_4053ins328), in exon 4 of *RP1* has been reported in Japanese and Korean patients with RP [38–40]. The different inheritance pattern likely depends on the location of the variant, because truncating variants in the middle of *RP1* (c.1981-c.2749) are most likely to be dominant [35]. Furthermore, as the insertion occurs in the last exon of the NM_006269.2 transcript encoded by the *RP1* gene, it is likely to escape nonsense-mediated decay [41]. However, it remains to be studied if the *RP1* mRNA transcripts harboring the L1 insertion are being translated into a truncated RP1 of 705 amino acids in the patients. It has been previously suggested that heterozygous truncating mutations resulting in RP1 of 677-917 amino acids may cause dominant RP via dominant negative effect [42]. The biological function of another RP1 isoform (NP_001362583.1), encoded by transcript variant NM_001375654.1, is currently not known. It should be noted however that this transcript variant lacks the exon 4 of NM_006269.2 and should thus not be affected by the L1 insertion.

Alterations from multiple TEs, most notably from L1, Alu, and SINE-VNTR-Alu (SVA) insertions, have more generally been described as the underlying genetic cause in patients with IRDs, such as insertion of an SVA F retrotransposon in the Bardet-Biedl syndrome 1 (*BBS1*) gene in patients with BBS, and an intronic SVA insertion in the major facilitator superfamily domain containing 8 (*MFSD8*) gene in a patient with CLN7 form of the Batten disease [11, 12]. An intronic L1 insertion in the *RP2* gene has been described in a patient with X-linked RP, and an Alu insertion in the male germ cell-associated kinase (*MAK*) gene in several RP patients of Ashkenazi Jewish ancestry [13–16]. Furthermore, previous large-scale exome sequencing studies have reported that 0.03-0.04% of diseases are likely caused by mobile element insertions in the coding sequences of genes [21, 22, 43–45]. In line with these reports, the *RP1* c.2106_2107insL1 identified in this study is an unexpected, but not an unprecedented finding most likely describing a further IRD caused by a transposable element.

While several tools for identifying TEs from short-read NGS data are available, they are easily missed unless specifically looked for (for review, see [46]). Also, the characterisation of several kb long elements is impossible from the short reads. Indeed, the identified LINE-1 insertion was originally missed by diagnostic gene panels, and even after reanalysis of the data, its size remained unknown.

Long-read sequencing techniques, including nanopore and single-molecule real-time (SMRT) sequencing, show promise in the identification and description of complex SVs [23, 24, 47]. Targeted adaptive nanopore sequencing allowed us to determine the size, sequence, and breakpoints of the 5.6 kb L1 insertion. Similarly, nanopore sequencing has been used to characterise likely pathogenic SVs in the eyes shut homolog (*EYS*) gene in two Japanese patients with RP of otherwise unresolved origin [6]. Furthermore, nanopore sequencing deciphered pathogenic SVs in centrosomal protein 78 (*CEP78*) in patients with autosomal recessive cone-rod dystrophy with hearing loss [7], and SMRT sequencing has characterised pathogenic variants, including SVs, within the highly complex opsin L and M encoding *OPN1LW/OPN1MW* gene cluster from patients with colour vision impairment [48]. It should be noted that in a retrospective analysis of our nanopore sequencing data using Sniffles2 structural variant caller [49], we could detect the L1 insertion. However, without prior indication of the location, it could have been difficult to filter out the variant of interest from the thousands of called structural variants.

In addition to the targeted adaptive nanopore sequencing described here, an alternative approach to detect mobile element insertions using nanopore sequencing utilizes long-range PCR amplification of the target gene locus before sequencing [50]. This has the benefit of requiring less input DNA and sequencing of only the specific PCR products (i.e., the amplified target gene). However, it requires prior knowledge of the location of the insert for designing primers, additional laboratory work, and may create challenges for the data analysis [51, 52]. On the other hand, the drawback of targeted adaptive nanopore sequencing is the reduced performance of the flow cell over time. This is due to the adaptive mode where the movement of a DNA molecule through the nanopores is reversed if the sequence is not included in the target panel. As such, the method is not optimal for sequencing a single target gene since most of the reads would be rejected during the adaptive sequencing mode. However, this approach has the benefit of not requiring additional handling of the DNA samples before sequencing. You simply define the desired target regions in a .bed file.

In conclusion, given that CNVs contribute to IRD in an estimated 9% of cases [10], more thorough analysis of genomic regions of IRD-associated genes should be considered whenever conventional NGS analysis is uninformative.

## Data Availability

The data from this project are available from the corresponding author upon reasonable request.

## Supplementary Materials

Supplementary 1 File S1: primers and PCR conditions used in the study.

Supplementary 2 File S2: sequences and alignments of the identified insertion.

Supplementary 3 Table S1: genetic positions and overlapping genes included in the custom target panel for targeted adaptive nanopore sequencing.
