# Supplementary material for: Characterisation of a LINE-1 Insertion in the RP1 Gene by Targeted Adaptive Nanopore Sequencing in a Family with Retinitis Pigmentosa
Source: Hum Mutat. 2024 Feb 9;2024:6580561. doi: 10.1155/2024/6580561 (PMC11919084; doi:10.1155/2024/6580561)
Supplement: Supplementary 1 — File S1: primers and PCR conditions used in the study. [file 6580561.f1.pdf]

Supplementary File S1

The primers used for the PCR amplification of the predicted 64bp L1 insert and the L1 insert breakpoints identified by nanopore sequencing:

**Description**	**Amplicon**	**Forward primer 5’–3’**	**Reverse primer 5’–3’**
Predicted 64bp L1 insert	5’ breakpoint	CATCCTCTACTGTCACTGCAAGA	AGAGACTTAGACTCCCACACA
	3’ breakpoint	TGTGTGGGAGTCTAAGTCTCT	GCACTTGCTTTCACTTTATCCCC
Nanopore L1 insert	5’ breakpoint	Same as for the 64bp 5’ breakpoint	Same as for the 64bp 5’ breakpoint
	3’ breakpoint	TTTGGATTTTGGTTTTCTTGC	TGGAAATCGTGGAATTGAGA

The PCR cycling conditions for the 5’-breakpoint and for the initially predicted 3’ breakpoint of the 64bp L1 insert were as follows:

Temperature	Duration	
94°C	10 min	
94°C	45 s	35 cycles
52°C	45 s	
72°C	60 s	
72°C	10 min	

The 3’-breakpoint identified by nanopore sequencing was amplified using touchdown PCR. The cycling conditions for the 3’-breakpoint were as follows:

Temperature	Duration	
94°C	10 min	
94°C	45 s	10 cycles
64°C -1°/cycle	45 s	
52°C	45 s	
72°C	60 s	
94°C	45 s	25 cycles
54°C	45 s	
72°C	45 s	
72°C	5 min	

The purified PCR products were prepared for sanger sequencing using BigDye Terminator v3.1 Cycle Sequencing Kit (Applied Biosystems, Thermo Fisher Scientific, Waltham, Massachusetts, USA) in 10 µl reaction volumes according to manufacturer’s instructions with the following cycling parameters:

Temperature	Duration	
96°C	60 s	
96°C	10 s	29 cycles
52°C	10 s	
60°C	4 min	
