# Supplementary material for: Characterisation of a LINE-1 Insertion in the RP1 Gene by Targeted Adaptive Nanopore Sequencing in a Family with Retinitis Pigmentosa
Source: Hum Mutat. 2024 Feb 9;2024:6580561. doi: 10.1155/2024/6580561 (PMC11919084; doi:10.1155/2024/6580561)
Supplement: Supplementary 2 — File S2: sequences and alignments of the identified insertion. [file 6580561.f2.pdf]

Supplementary File S2

Sequences of the LINE-1 insertions in exon 4 of *RP1* fully covered by targeted adaptive nanopore sequencing from patients III.1 and III.2.

```
>Patient_III_1_sequence_1
GTGGGGTGTTAAAGTCTCCCATTATTAATGGGAGTCTAAGTCTCTTTGTAGGTCACTGAGGACTTGCTTTA
TGAATCTGGGTGCTCCTGTATTGGGTGCATAAATATTTAGGATAGTTAGCTCCTCTTGTTGAATTGATCCC
TTTACCATTATGTAATGGCCTTCTTTGTCTCTTTTGATCTTTGTTGGTTTAAAGTCTGTTTTATCAGAGAC
TAGGATTGCAACCCCTGCCTTTTTTTGTTTTCCATTGGCTTGGTAGATCTTCCTCCATCCTTTTATTTTGA
GCCTATGTGTGTCTCTGCACGTGAGATAAATGGAATACAGCACACTGATGGGTCTTGACTCTTTATCCAAC
TTGCCAGTCTGTGTCTTTTAATTGCAGAATTTAGTCCATTTATATTTAAAGTTAATATTGTTATGTGTGAA
TTTGATCCTGTCATTATGATGTTAGCTGGTGATTTTGCTCATTAGTTGATGCAGTTTCTTCCTAGTCTCGA
TGGTCTTTACATTTTGGCATGATTTTGCAGCGGCTGGTACCGGTTGTTCCTTTCCATGTTTAGCGCTTCCT
TCAGGAGCTCTTTTAGGGCAGGCCTGGTGGTGACAAAATCTCTCAACATTTGCTTGTCTATAAAGTATTTT
ATTTCTCCTTCACTTATGAAGCTTAGTTTGGCTGGATATGAAATTCTGGGTTGAAAATTCTTTTCTTTAAG
AATGTTGAATATTGGCCCCCACTCTCTTCTGGCTTGTAGGGTTTCTGCCGAGAGATCCGCTGTTAGTCTGA
TGGGCTTTCTGAGGGTAACCTTCCCTTTGCTTGGCTGCCCTTAACATTTTTTCCTTCATTTCAACTTTGGT
GAATCTGACAATTATGTGTCTTGGAGTTGCTCTTCTCGAGGAGTATCTTTGTGGCGTTCTCTGTATTTCCT
GAATCTGAACGTTGGCCTGCCTTGCTAGATTGGGGAAGTTCTCCTGGATAATATCCTGCAGAGTGTTTTCC
AACTTGGTTCCATTCTCCACATCACTTTCAGGTACACCAATCAGACGTAGATTTGGTCTTTTCACATAGTC
CCATATTTCTTGGAGGCTTTGCTCATTTCTTTTTATTCTTTTTTCTCTAAACTTCCCTTCTCGCTTCATTT
CATTCATTTCATCTTCCATTGCTGATACCCTTTCTTCCAGTTGATCGGCTCCTGAGGCTTGTGCATTCTTC
ACGTAGTTCTCGAGCCTTGGTTTTCAGCTCCATCAGCTCCTTTAAGCACTTCTCTGTATTGGTTATTCTAG
TTATACATTCTTCTAAATTTTTTTCAAAGTTTTCAACTTCTTTGCCTTTGGTTTGAATGTCCTCCCGTAGC
TCAGAGTAATTTGATCGTCTGAAGCCTTCTTCTCTCAGCTCATCAAAATCATTCTCCATCCAGCTTTGTTC
TGTTGCTGGTGAGGAACTGCGTTCCTTTGGAGGAGGAGAGGCGCTCTGCGTTTTAGAGTTTCCAGTTTTTC
TGTTCTGTTTTTTCCCCATCTTTGTGGTTTTATCTACTTTTGGTCTTTGATGATGGTGATGTACAGATGGG
TTTTTGGTGTAGATGTCCTTTCTGGTTGTTAGTTTTCCTTCTAACAGACAGGACTTTTAACACTCCACTGT
CAACATTAGACAGATCAACGAGATAGAAAGTCAACAAGGATACCCAGGAATTGAACTCAGCTCTGCACCAA
GCAGACCTAATAGACATCTACAGAACTCTCCACCCCAAATCAACAGAATATACATTTTTTTCAGCACCACA
CCACACCTATTCCAAAATTGACCACATAGTTGGAAGTAAAGCTCTCCTCAGCAAATGTAAAAGAACAGAAA
TTATAACAAACTATCTCTCAGACCACAGTGCAATCAAACTAGAACTCAGGATTAAGAATCTCACTCAAAGC
CGCTCAACTACATGGAAACTGAACAACCTGCTCCTGAATGACTACTGGGTACATAACGAAATGAAGGCAGA
AATAAAGATGTTCTTTGAAACCAACGAGAACAAAGACACCACATACCAGAATCTCTGGGACGCATTCAAAG
CAGTGTGTAGAGGGAAATTTATAGCACTAAATGCCTAAGCAAGAGAAAGCAGGAAAGATCCAAAATTGACA
CCCTAACATCACAATTAAAAGAACTAGAAAAGCAAGAGCAAACACATTCAAAAGCTAGCAGAAGGCAAGAA
ATAACTAAAATCAGAGCAGAACTGAAGGAAATAGAGACACAAAAAACCCTTCAAAAAATCAATGAATCCAG
GAGCTGGTTTTTTGAAAGGATCAAAAAATTGATAGACCGCTAGCAAGACTAATAAAGAAAAAAAGAGAGAA
GAATCAAATAGACACAATAAAAAATGATAAAGGGGATATCACCACCGATCCCACAGAAATACAAACTACCA
TCAGAGAATACTACAAACACCTCTACGCAAATAAACTAGAAAATCTAGAAGAAATGGATACATTCCTCGAC
ACATACACTCTCCCAAGACTAAACCAGGAAGAAGTTGAATCTCTGAATCGATCAATAACAGGCTCTGAAAT
TGTGGCAATAATCAATAGTTTACCAACCAAAAAGAGTCCAGGACCAGATGGATTCACAGCCGAATTCTACC
AGAGGTACAAGGAGGAACTGGTACCATTCCTTCTGAAACTATTCCAATCAATAGAAAAAGAGGGAATCCTC
CCTAACTCATTTTATGAGGCCAGCATCATTCTGATACCAAAGCCGGGCAGAGACACAACCAAAAAAGAGAA
TTTTAGACCAATATCCTTGATGAACATTGATGCAAAAATCCTCAATAAAATACTGGCAAACCGAATCCAGC
ACATCAAAAAGCTTATCCACCATGATCAAGTGGGCTTCATCCCTGGGATGCAAGGCTGGTTCAATATACGC
AAATCAATAAATGTAATCCAGCATATAAACAGAGCCAAAGACAAAAACCACATGATTATCTCAATAGATGC
AGAAAAAGCCTTTGACAAAATTCAACAACCCTTCATGCTAAAAACTCTCAATAAATTAGGTATTGATGGGA
CGTATTTCAAAATAATAAGAGCTATCTATGACAAACCCACAGCCAATATCATACTGAATGGGCAAAAACTG
GAAGCATTCCCTTTGAAAACCGGCACAAGACAGGGATGCCCTCTCTCACCGCTCCTATTCAACATAGTGTT
GGAAGTTCTGGCCAGGGCAATCAGGCAGGAGAAGGAAATAAAGGGTATTCAATTAGGAAAAGAGGAAGTCA
AATTGTCCCTGTTTGCAGACGACATGATTGTTTATCTAGAAAACCCCATCGTCTCAGCCCAAAATCTCCTT
AAGCTGATAAGCAACTTCAGCAAAGTCTCAGGATACAAAATCAATGTACAAAAATCACAAGCATTCTTATA
CACCAACAACAGACAAACAGAGAGCCAAATCATGGGTGAACTCCCATTCACAATTGCTTCAAAGAGAATAA
AATACCTAGGAATCCAACTTACAAGGGATGTGAAGGACCTCTTCAAGGAGAACTACAAACCACTGCTCAAG
GAAATAAAAGAGGAGACAAACAAATGGAAGAACATTCCATGCTCATGGGTAGGAAGAATCAATATCGTGAA
```

AATGGCCATACTGCCCAAGGTAATTTACAGATTCAATGCCATCCCCATCAAGCTACCAATGACTTTCTTCA
CAGAATTGGAAAAAACTACTTTAAAGTTCATATGGAACCAAAAAAGAGCCCGCATTGCCAAGTCAATCCTA
AGCCAAAAGAACAAAGCTGGAGGCATCACACTACCTGACTTCAAACTATACTACAAGGCTACAGTAACCAA
AACAGCATGGTACTGGTACCAAAACAGAGATATAGATCAATGGAACAGAACAGAGCCCTCAGAAATAATGC
CGCATATCTACAACTATCTGATCTTTGACAAACCTGAGAAAAACAAGCAATGGGGAAAGGATTCCCTATTT
AATAAATGGTGCTGGGAAAACTGGCTAGCCATATGTAGAAAGCTGAAACTGGATCCCTTCCTTACACCTTA
TACAAAAATCAATTCAAGATGGATTAAAGATTTAAACGTTAAACTCAAAACCATAAAAACCCTAGAAGAAA
ACCTAGGCATTACCATTCAGGACATAGGCGTGGGCAAGGACTTCATGTCCAAAACACCAAAAGCAATGGCA
ACAAAAGACAAAATTGACAAATGGGATCTAATTAAACTAAAGAGCTTCTGCACAGCAAAAGAAACTACCAT
CAGAGTGAACAGGCAACCTACAACATGGGAGAAAATTTTTGCAACCTACTCATCTGACAAAGGGCTAATAT
CCAGAATCTACAATGAACTCAAACAAATTTACAAGAAAAAAACAAACAACCCCATCAAAAAGTGGGCGAAG
GACATGAACAGACACTTCTCAAAAGAAGACATTTATGCAGCCAAAAAACACATGAAGAAATGCTCATCATC
ACTGGCCATCAGAGAAATGCAAATCAAAACCACTATGAGATATCATCTCACACCAGTTAGAATGGCAATCA
TTAAAAAGTCAGGAAACAACAGGTGCTGGAGAGGATGCGGAGAAATAGGAACACTTTTACACTGTTGGTGG
GACTGTAAACTAGTTCAACCATTGTGGAAGTCAGTGTGGCGATTCCTCAGGGATCTAGAACTAGAAATACC
ATTTGACCCAGCCATCCCATTACTGGGTATATACCCAAATGAGTATAAATCATGCTGCTATAAAGACACAT
GCACACGTATGTTTATTGCGGCACTATTCACAATAGCAAAGACTTGGAACCAACCCAAATGTCCAACAATG
ATAGACTGGATTAAGAAAATGTGGCACATATACACCATGGAATACTATGCAGCCATAAAAAATGATGAGTT
CATATCCTTTGTAGGGACATGGATGAAATTGGAAACCATCATTCTCAGCAAACTATCGCAAGAACAAAAAA
CCAAACACCGCATATTCTCACTCATAGGTGGGAATTGAACAATGAGATCACATGGACCCAGGAAGGGGAAC
ATCACACTCTGGGGACTGTGGTGGGCCGGGGGAGGGGGGAGGGATAGCATTGGGAGATATACCTAATGCTA
GATGACACATTAGTGGGTGCAGCGCACCAGCATGGCACATGTATACATATGTAACTAACCTGCACAATGTG
CACATGTACCCTAAAACTTAGAGTATAATAAAAAAAAAAAAAAAAAAAAATAGAAAGAAAGAAATAAATAAAA
AAAGAAAAGAAAGAAAGAAAGAAAGAAAGAAAGAAAGAAAGAAAGAAAGAAAGAAAGAAAGAAAGAAAGAA
AGAAAGAAAGAAAGAAAGAGAAAAGCAAAATGTCTATTCCGGCTCATTGCCTATTTTTTGAATTTGGATTT
TGTTTTCTTGCTATTGAGTTGTTTGAGTTCTTTATATATTTTGAGTAATAATCCCTTATCAGATGTATGCT
TTGCAAATAAATTTTCCATTCCATGCAAAAAAAAAAAAAAAAAAAAAAAAAAAAAAAAAAAAAAAAGAATG
AGAGAATA

>Patient_III_2_sequence_1
GTGGGGTGTTAAAGTCTCCCATTATTAATGTGTGGGAGTCTAAGTCTCTTTGTAGGTCACTGAGGACTTGC
TTTATGAATCTGGGTGCTCCTGTATTGGGTGCATAAATATTTAGGATAGTTAGCTCCTCTTGTTGAATTGA
TCCCTTTACCATTATGTAATGGCCTTCTTTGTCTCTTTTGATCTTTGTTGGTTTAAAGTCTGTTTTATCAG
AGACTAGGATTGCAACCCCTGCCTTTTTTTGTTTTCCATTGGCTTGGTAGATCTTCCTCCATCCTTTTATT
TTGAGCCTATGTGTGTCTCTGCACGTGAGATGGGTTTCCTGAATACAGCACACTGATGGGTCTTGACTCTT
TATCCAACTTGCCAGTCTGTGTCTTTTAATTGCAGAATTTAGTCCATTTATATTTAAAGTTAATATTGTTA
TGTGTGAATTTGATCCTGTCATTATGATGTTAGCTGGTGATTTTGCTCATTAGTTGATGCAGTTTCTTCCT
AGTCTCGATGGTCTTTACATTTTGGCATGATTTTGCAGCGGCTGGTACCGGTTGTTCCTTTCCATGTTTAG
CGCTTCCTTCAGGAGCTCTTTTAGGGCAGGCCTGGTGGTGACAAAATCTCTCAACATTTGCTTGTCTATAA
AGTATTTTATTTCTCCTTCACTTATGAAGCTTAGTTTGGCTGGATATGAAATTCTGGGTTGAAAATTCTTT
TCTTTAAGAATGTTGAATATTGGTCCCCACTCTCTTCTGGCTTGTAGGGTTTCTGCCGAGAGATCCGCTGT
TAGTCTGATGGGCTTTCCTTTGAGGGTAACCCGACCTTTCTCTCTGGCTGCCCTTAACATTTTTTCCTTCA
TTTCAACTTTGGTGAATCTGACAATTATGTGTCTTGGAGTTGTTCTTCTCGAGGAGTATCTTTGTGGCGTT
CTCTGTATTTCCTGAATCTGAACGTTGGCCTGCCTTGCTAGATTGGGGAAGTTCTCCTGGATAATATCCTG
CAGAGTGTTTTCCAACTTGGTTCCATTCTCCACATCACTTTCAGGTACACCAATCAGACGTAGATTTGGTC
TTTTCACATAGTCCCATATTTCTTGGAGGCTTTGCTCATTTCTTTTTATTCTTTTTTCTCTAAACTTCCCT
TCTCGCTTCATTTCATTCATTTCATCTTCCATTGCTGATACCCTTTCTTCCAGTTGATCGCATCGGCTCCT
GAGGCTTCTGCATTCTTCACGTAGTTCTCGAGCCTTGGTTTTCAGCTCCATCAGCTCCTTTAAGCACTTCT
CTGTATTGGTTATTCTAGTTATACATTCTTCTAAATTTTTTTCAAAGTTTTCAACTTCTTTGCCTTTGGTT
TGAATGTCCTCCCGTAGCTCAGAGTAATTTGATCGTCTGAAGCCTTCTTCTCTCAGCTCATCAAAATCATT
CTCCATCCAGCTTTGTTCTGTTGCTGGTGAGGAACTGCGTTCCTTTGGAGGAGGAGAGGCGCTCTGCGTTT
TAGAGTTTCCAGTTTTTCTGTTCTGTTTTTTCCCCATCTTTGTGGTTTTATCTACTTTTGGTCTTTGATGA
TGGTGATGTACAGATGGGTTTTCGGTGTAGATGTCCTTTCTGGTTGTTAGTTTTCCTTCTAACAGACAGGA
CCCTTTAACACCCCACTGTCAACATTAGACAGATCAACGAGACAGAAAGTCAACAAGGATACCCAGGAATT
GAACTCAGCTCTGCACCAAGCAGACCTAATAGACATCTACAGAACTCTCCACCCCAAATCAACAGAATATA
CATTTTTTTCAGCACCACACCACACCTATTCCAAAATTGACCACATAGTTGGAAGTAAAGCTCTCCTCAGC
AAATGTAAAAGAACAGAAATTATAACAAACTATCTCTCAGACCACAGTGCAATCAAACTAGAACTCAGGAT
TAAGAATCTCACTCAAAGCCGCTCAACTACATGGAAACTGAACAACCTGCTCCTGAATGACTACTGGGTAC

ATAACGAAATGAAGGCAGAAATAAAGATGTTCTTTGAAACCAACGAGAACAAAGACACCACATACCAGAAT
CTCTGGGACGCATTCAAAGCAGTGTGTAGAGGGAAATTTATAGCACTAAATGCCTAAAAGAGAAAGCAGGA
AAGATCCAAAATTGACACCCTAACATCACAATTAAAAGAACTAGAAAAGCAAGAGCAAACACATTCAAAAG
CTAGCAGAAGGCAAGAAATAACTAAAATCAGAGCAGAACTGAAGGAAATAGAGACACAAAAAACCCTTCAA
AAAAATCAATGAATCCAGGAGCTGGTTTTTTGAAAGGATCAACAAAATTGATAGACCGCTAGCAAGACTAA
TAAAGAAAAAAAGAGAGAAGAATCAAATAGACACAATAAAAAATGATAAAGGGGATATCACCACCGATCCC
ACAGAAATACAAACTACCATCAGAGAATACTACAAACACCTCTACGCAAATAAACTAGAAAATCTAGAAGA
AATGGATACATTCCTCGACACATACACTCTCCCAAGACTAAACCAGGAAGAAGTTGAATCTCTGAATCGAC
CAATAACAGGCTCTGAAATTGTGGCAATAATCAATAGTTTACCAACCAAAAAGAGTCCAGGACCAGATGGA
TTCACAGCCGAATTCTACCAGAGGTACAAGGAGGAACTGGTACCATTCCTTCTGAAACTATTCCAATCAAT
AGAAAAAGAGGGAATCCTCCCTAACTCATTTTATGAGGCCAGCATCATTCTGATACCAAAGCCGGGCAGAG
ACACAACCAAAAAAGAGAATTTTAGACCAATATCCTTGATGAACATTGATGCAAAAATCCTCAATAAAATA
CTGGCAAACCGAATCCAGCAGCACATCAAAAAGCTTATCCACCATGATCAAGTGGGCTTCATCCCCTGGGA
TGCAAGGCTGGTTCAATATACGCAAATCAATGTAATCCAGCATATAAACAGAGCCAAAGACAAAAACCACA
TGATTATCTCAATAGATGCAGAAAAAGCCTTTGACAAAATTCAACAACCCTTCATGCTAAAAACTCTCAAT
AAATTAGGTATTGATGGGACGTATTTCAAAATAATAAGAGCTATCTATGACAAACCCACAGCCAATATCAT
ACTGAATGGGCAAAAACTGGAAGCATTCCCTTTGAAAACCGGCACAAGACAGGGATGCCCTCTCTCACCGC
TCCTATTCAACATAGTGTTGGAAGTTCTGGCCAGGGCAATCAGGCAGGAGAAGGAAATAAAGGGTATTCAA
TTAGGAAAAGAGGAAGTCAAATTGTCCCTGTTTGCAGACGACATGATTGTTTATCTAGAAAACCCCATCGT
CTCAGCCCAAAATCTCCTTAAGCTGATAAGCAACTTCAGCAAAGTCTCAGGATACAAAATCAATGTACAAA
AATCACAAGCATTCTTATACACCAACAACAGACAAACAGAGAGCCAAATCATGAGTGAACTCCCATTCACA
ATTGCTTCAAAGAGAATAAAATACCTAGGAATCCAACTTACAAGGGATGTGAAGGACCTCTTCAAGGAGAA
CTACAAACCACTGCTCAAGGAAATAAAAGAGGAGACAAACAAATGGAAGAACATTCCATGCTCATGGGTAG
GAAGAATCAATATCGTGAAAATGGCCATACTGCCCAAGGTAATTTACAGATTCAATGCCATCCCCATCAAG
CTACCAATGACTTTCTTCACAGAATTGGAAAAAACTACTTTAAAGTTCATATGGAACCAAAAAAGAGCCCG
CATTGCCAAGTCAATCCTAAGCCAAAAGAACAAAGCTGGAGGCATCACACTACCTGACTTCAAACTATACT
ACAAGGCTACAGTAACCAAAACAGCATGGTACTGGTACCAAAACAGAGATATAGATCAATGGAACAGAACA
GAGCCCTCAGAAATAATGCCGCATATCTACAACTATCTGATCTTTGACAAACCTGAGAAAAACAAGCAATG
GGGAAAGGATTCCCTATTTAATAAATGGTGCTGGGAAAACTGGCTAGCCATATGTAGAAAGCTGAAACTGG
ATCCCTTCCTTACACCTTATACAAAAATCAATTCAAGATGGATTAAAGATTTAAACGTTAAACCTAAAACC
ATAAAAACCCTAGAAGAAAACCTAGGCATTACCATTCAGGACATAGGCGTGGGCAAGGACTTCATGTCCAA
AACACCAAAAGCAATGGCAACAAAAGACAAAATTGACAAATGGGATCTAATTAAACTAAAGAGCTTCTGCA
CAGCAAAAGAAACTACCATCAGAGTGAACAGGCAACCTACAACATGGGAGAAAATTTTTGCAACCTACTCA
TCTGACAAAGGGCTAATATCCAGAATCTACAATGAACTCAAACAAATTTACAAGAAAAAAACAAACAACCC
CATCAAAAAGTGGGCGAAGGACATGAACAGACACTTCTCAAAAGAAGACATTTATGCAGCCAAAAAACACA
TGAAGAAATGCTCATCATCACTGGCCATCAGAGAAATGCAAATCAAAACCACTATGAGATATCATCTCACA
CCAGTTAGAATGGCAATCATTAAAAAGTCAGGAAACAACAGGTGCTGGAGAGGATGCGGAGAAATAGGAAC
ACTTTTACACTGTTGGTGGGACTGTAAACTAGTTCAACCATTGTGGAAGTCAGTGTGGCGATTCCTCAGGG
ATCTAGAACTAGAAATACCATTTGACCCAGCCATCCCATTACTGGGTATATACCCAAATGAGTATAAATCA
TGCTGCTATAAAGACACATGCACACGTATGTTTATTGCGGCACTATTCACAATAGCAAAGACTTGGAACCA
ACCCAAATGTCCAACAATGATAGACTGGATTAAGAAAATGTGGCACATATACACCATGGAATACTATGCAG
CCATAAAAAATGATGAGTTCATATCCTTTGTAGGGACATGGATGAAAATTGGAAATCATCATTCTCAGTAA
ACTATCGCAAGAACAAAAAACCAAACACCGCATATTCTCACTCATAGGTGGGAATTGAACAATGAGATCAC
ATGGACCCAGGAAGGGGAATATCACACTCTGGGGACTGTGGTGGGGTCGGGGAGGGGGGAGGGATAGCATT
GGGAGATATACCTAATGCTAGATGACACATTAGTGGGTGCAGCGCACCAGCATGGCACATGTATACATATG
TAACTAACCTGCACAATGTGCACATGTACCCTAAAACTTAGAGTATAATAAAAAAAAAAAAAAAAAAAATA
GAAAGAAAGAAATAAATAAAAAGAAAGAAAGAAAGAAAGAAAGAAAGAAAGAAAGAAAGAAAGAAAGAAAG
AAAGAAAGAAAGAAAGAAAGAAAGAAAGAAAGAAAGAAAGAGAAAAGCAAAATGTCTATTCCGGTCTATTG
CCTATTTTTTGAATTTGGATTTTGGTTTTCTTGCTATTGAGTTGTTTGAGTTCCTTATATATTTTGAGTAA
TAACTCCTTATCAGATGTATGCTTTGCAAATAAATTTCCCATTCCATGCAAAAAAAAAAAAAAAAAAAAAA
AAAAAAAAAAAAGAATGAGAGAATA

>Patient_III_2_sequence_2
GTGGTGTTAAAGTCTCCCATTATTAATGTGTGGGAGTCTAAGTCTCTTTGTAGGTCACTGAGGACTTGCTT
TATGAATCTGGGTGCTCCTGTATTGGGTGCATAAATATTTAGGATAGTTAGCTCCTCTTGTTGAATTGATC
CCTTTACCATTATGTAATGGCCTTCTTTGTCTCTTTTGATCTTTGTTGGTTTAAAGTCTGTTTTATCAGAG
ACTAGGATTGCAACCCCTGCCTTTTTTTGTTTTCCATTGGCTTGGTAGATCTTCCTCCATCCTTTTATTTT
GAGCCTATGTGTGTCTCTGCACGTGAGATGGGTTTCCTGAATACAGCACACTGATGGGTCTTGACTCTTTA

TCCAACTTGCCAGTCTGTGTCTTTTAATTGCAGAATTTAGTCCATTTATATTTAAAGTTAATATTGTTATG
TGTGAATTTGATCCTGTCATTATGATGTTAGCTGGTGATTTTGCTCATTAGTTGATGCAGTTTCTTCCTAG
TCTCGATGGTCTTTACATTTTGGCATGATTTTGCAGCGGCTGGTACCGGTTGTTCCTTTCCATGTTTAGCG
CTTCCTTCAGGAGCTCTTTTAGGGCAGGCCTGGTGGTGACAAAATCTCTCAACATTTGCTTGTCTATAAAG
TATTTTATTTCTCCTTCACTTATGAAGCTTAGTTTGGCTGGATATGAAATTCTGGGTTGAAAATTCTTTTC
TTTAAGAATGTTGAATATTGGCCCCCACTCTCTTCTGGCTTGTAGGGTTTCTGCCGAGAGATCCGCTGTTA
GTCTGATGGGCTTTCCTTTGAGGGTAACCCGACCTTTCTCTCTGGCTGCCCTTAACATTTTTTCCTTCATT
TCAACTTTGGTGAATCTGACAATTATGTGTCTTGGAGTTGCTCTTCTCGAGGAGTATCTTTGTGGCGTTCT
CTGTATTTCCTGAATCTGAACGTTGGCCTGCCTTGCTAGATTGGGGAAGTTCTCCTGGATAATATCCTGCA
GAGTGTTTTCCAACTTGGTTCCATTCTCCACATCACTTTCAGGTACACCAATCAGACGTAGATTTGGTCTT
TTCACATAGTCCCATATTTCTTGGAGGCTTTGCTCATTTCTTTTTATTCTTTTTTCTCTAAACTTCCCTTC
TCGCTTCATTTCATTCATTTCATCTTCCATTGCTGATACCCTTTCTTCCAGTTGATCGCATCGGCTCCTGA
GGCTTCTGCATTCTTCACGTAGTTCTCGAGCCTTGGTTTTCAGCTCCATCAGCTCCTTTAAGCACTTCTCT
GTATTGGTTATTCTAGTTATACATTCTTCTAAATTTTTTTCAAAGTTTTCAACTTCTTTGCCTTTGGTTTG
AATGTCCTCCCGTAGCTCAGAGTAATTTGATCGTCTGAAGCCTTCTTCTCTCAGCTCATCAAAATCATTCT
CCATCCAGCTTTGTTCTGTTGCTGGTGAGGAACTGCGTTCCTTTGGAGGAGGAGAGGCGCTCTGCGTTTTA
GAGTTTCCAGTTTTTCTGTTCTGTTTTTTCCCCATCTTTGTGGTTTTATCTACTTTTGGTCTTTGATGATG
GTGATGTACAGATGGGTTTTCGGTGTAGATGTCCTTTCTGGTTGTTAGTTTTCCTTCTAACAGACAGGACC
CTTTAACACCCCACTGTCAACATTAGACAGATCAACGAGACAGAAAGTCAACAAGGATACCCAGGAATTGA
ACTCAGCTCTGCACCAAGCAGACCTAATAGACATCTACAGAACTCTCCACCCCAAATCAACAGAATATACA
TTTTTTTCAGCACCACACCACACCTATTCCAAAATTGACCACATAGTTGGAAGTAAAGCTCTCCTCAGCAA
ATGTAAAAGAACAGAAATTATAACAAACTATCTCTCAGACCACAGTGCAATCAAACTAGAACTCAGGATTA
AGAATCTCACTCAAAGCCGCTCAACTACATGGAAACTGAACAACCTGCTCCTGAATGACTACTGGGTACAT
AACGAAATGAAGGCAGAAATAAAGATGTTCTTTGAAACCAACGAGAACAAAGACACCACATACCAGAATCT
CTGGGACGCATTCAAAGCAGTGTGTAGAGGGAAATTTATAGCACTAAATGCCTACAAGAGAAAGCAGGAAA
GATCCAAAATTGACACCCTAACATCACAATTAAAAGAACTAGAAAAGCAAGAGCAAACACATTCAAAAGCT
AGCAGAAGGCAAGAAATAACTAAAATCAGAGCAGAACTGAAGGAAATAGAGACACAAAAAACCCTTCAAAA
AATCAATGAATCCAGGAGCTGGTTTTTTGAAAGGATCAACAAAATTGATAGACCGCTAGCAAGACTAATAA
AGAAAAAAAGAGAGAAGAATCAAATAGACACAATAAAAAATGATAAAGGGGATATCACCACCGATCCCACA
GAAATACAAACTACCATCAGAGAATACTACAAACACCTCTACGCAAATAAACTAGAAAATCTAGAAGAAAT
GGATACATTCCTCGACACATACACTCTCCCAAGACTAAACCAGGAAGAAGTTGAATCTCTGAATCGACCAA
TAACAGGCTCTGAAATTGTGGCAATAATCAATAGTTTACCAACCAAAAAGAGTCCAGGACCAGATGGATTC
ACAGCCGAATTCTACCAGAGGTACAAGGAGGAACTGGTACCATTCCTTCTGAAACTATTCCAATCAATAGA
AAAAGAGGGAATCCTCCCTAACTCATTTTATGAGGCCAGCATCATTCTGATACCAAAGCCGGGCAGAGACA
CAACCAAAAAAGAGAATTTTAGACCAATATCCTTGATGAACATTGATGCAAAAATCCTCAATAAAATACTG
GCAAACCGAATCCAGCAGCACATCAAAAAGCTTATCCACCATGATCAAGTGGGCTTCATCCCTGGGATGCA
AGGCTGGTTCAATATACGCAAATCAATAAATGTAATCCAGCATATAAACAGAGCCAAAGACAAAAACCACA
TGATTATCTCAATAGATGCAGAAAAGCCTTTGACAAAATTCAACAACCCTTCATGCTAAAAACTCTCAATA
AATTAGGTATTGATGGGACGTACTTCAAAATAATAAGAGCTATCTATGACAAACCCACAGCCAATATCATA
CTGAATGGGCAAAAACTGGAAGCATTCCCTTTGAAAACCGGCACAAGACAGGGATGCCCTCTCTCACCGCT
CCTATTCAACATAGTGTTGGAAGTTCTGGCCAGGGCAATCAGGCAGGAGAAGGAAATAAAGGGTATTCAAT
TAGGAAAAGAGGAAGTCAAATTGTCCCTGTTTGCAGACGACATGATTGTTTATCTAGAAAACCCCATCGTC
TCAGCCCAAAATCTCCTTAAGCTGATAAGCAACTTCAGCAAAGTCTCAGGATACAAAATCAATGTACAAAA
ATCACAAGCATTCTTATACACCAACAACAGACAAACAGAGAGCCAAATCATGGGTGAACTCCCATTCACAA
TTGCTTCAAAGAGAATAAAATACCTAGGAATCCAACTTACAAGGGATGTGAAGGACCTCTTCAAGGAGAAC
TACAAACCACTGCTCAAGGAAATAAAAGAGGAGACAAACAAATGGAAGAACATTCCATGCTCATGGGTAGG
AAGAATCAATATCGTGAAAATGGCCATACTGCCCAAGGTAATTTACAGATTCAATGCCATCCCCATCAAGC
TACCAATGACTTTCTTCACAGAATTGGAAAAAACTACTTTAAAGTTCATATGGAACCAAAAAAGAGCCCGC
ATTGCCAAGTCAATCCTAAGCCAAAAGAACAAAGCTGGAGGCATCACACTACCTGACTTCAAACTATACTA
CAAGGCTACAGTAACCAAAACAGCATGGTACTGGTACCAAAACAGAGATATAGATCAATGGAACAGAACAG
AGCCCTCAGAAATAATGCCGCATATCTACAACTATCTGATCTTTGACAAACCTGAGAAAAACAAGCAATGG
GGAAAGGATTCCCTATTTAATAAATGGTGCTGGGAAAACTGGCTAGCCATATGTAGAAAGCTGAAACTGGA
TCCCTTCCTTACACCTTATACAAAAATCAATTCAAGATGGATTAAAGATTTAAACGTTAAACCTAAAACCA
TAAAAACCCTAGAAGAAAACCTAGGCATTACCATTCAGGACATAGGCGTGGGCAAGGACTTCATGTCCAAA
ACACCAAAAGCAATGGCAACAAAAGACAAAATTGACAAATGGGATCTAATTAAACTAAAGAGCTTCTGCAC
AGCAAAAGAAACTACCATCAGAGTGAACAGGCAACCTACAACATGGGAGAAAATTTTTGCAACCTACTCAT
CTGACAAAGGGCTAATATCCAGAATCTACAATGAACTCAAACAAATTTACAAGAAAAAAACAAACAACCCC
ATCAAAAAGTGGGCGAAGGACATGAACAGACACTTCTCAAAAGAAGACATTTATGCAGCCAAAAAACACAT

```
GAAGAAATGCTCATCATCACTGGCCATCAGAGAAATGCAAATCAAAACCACTATGAGATATCATCTCACAC
CAGTTAGAATGGCAATCATTAAAAAGTCAGGAAACAACAGGTGCTGGAGAGGATGCGGAGAAATAGGAACA
CTTTTACACTGTTGGTGGGACTGTAAACTAGTTCAACCATTGTGGAAGTCAGTGTGGCGATTCCTCAGGGA
TCTAGAACTAGAAATACCATTTGACCCAGCCATCCCATTACTGGGTATATACCCAAATGAGTATAAATCAT
GCTGCTATAAAGACACATGCACACGTATGTTTATTGCGGCACTATTCACAATAGCAAAGACTTGGAACCAA
CCCAAATGTCCAACAATGATAGACTGGATTAAGAAAATGTGGCACATATACACCATGGAATACTATGCAGC
CATAAAAAATGATGAGTTCATATCCTTTGTAGGGACATGGATGAAATTGGAAACCATCATTCTCAGTAAAC
TATCGCAAGAACAAAAAACCAAACACCGCATATTCTCACTCATAGGTGGGAATTGAACAATGAGATCATGG
ACCCAGGAAGGGGAATATCACACTCTGGGGACTGGGGGTGGGGTCGGGGGAGGGGGGAGGGATAGCATTGG
GAGATATACCTAATGCTAGATGACACATTAGTGGGTGCAGCGCACCAGCATGGCACATGTATACATATGTA
ACTAACCTGCACAATGTGCACATGTACCCTAAAACTTAGAGTATAATAAAAAAAAAAAAAAAAAAAAATA
GAAAGAAAGAAATAAATAAAAAAGAAAGAAAGAAAGAAAGAAAGAAAGAAAGAAAGAAAGAAAGAAAGAAG
AAAGAAAGAAAGAAAGAAAGAAAGAAAGAAAGAAAGAAAGAGAAAAGAAATGTCTATTCAGTCTATTGCCT
ATTTTTTGGAATTTGGATTTTGGTTTTCTTGCTATTGAGTTGTTTGAGTTCCTTATATATTTTGAGTAATA
ACTCCTTATCAGATGTATGCTTTGCAAATAAATTTTCCCATTCCATGCAAAAAAAAAAAAAAAAAAAAAA
AAAAAAAAAAAAAAAAAAAAAAAAGAATGAGAGAATA
```

The fully covered LINE-1 insertions were aligned with MUSCLE multiple sequence alignment using Unipro UGENE (v.49.0).

```
III.1_1   GTGGGGTGTTAAAGTCTCCCATTATTAA----TGGGAGTCTAAGTCTCTTTGTAGGTCACTGAGGACTTG 70
III.2_1   GTGGGGTGTTAAAGTCTCCCATTATTAATGTGTGGGAGTCTAAGTCTCTTTGTAGGTCACTGAGGACTTG 70
III.2_2   GT--GGTGTTAAAGTCTCCCATTATTAATGTGTGGGAGTCTAAGTCTCTTTGTAGGTCACTGAGGACTTG 70
          **  ************************    **************************************

III.1_1   CTTTATGAATCTGGGTGCTCCTGTATTGGGTGCATAAATATTTAGGATAGTTAGCTCCTCTTGTTGAATT 140
III.2_1   CTTTATGAATCTGGGTGCTCCTGTATTGGGTGCATAAATATTTAGGATAGTTAGCTCCTCTTGTTGAATT 140
III.2_2   CTTTATGAATCTGGGTGCTCCTGTATTGGGTGCATAAATATTTAGGATAGTTAGCTCCTCTTGTTGAATT 140
          **********************************************************************

III.1_1   GATCCCTTTACCATTATGTAATGGCCTTCTTTGTCTCTTTTGATCTTTGTTGGTTTAAAGTCTGTTTTAT 210
III.2_1   GATCCCTTTACCATTATGTAATGGCCTTCTTTGTCTCTTTTGATCTTTGTTGGTTTAAAGTCTGTTTTAT 210
III.2_2   GATCCCTTTACCATTATGTAATGGCCTTCTTTGTCTCTTTTGATCTTTGTTGGTTTAAAGTCTGTTTTAT 210
          **********************************************************************

III.1_1   CAGAGACTAGGATTGCAACCCCTGCCTTTTTTTGTTTTCCATTGGCTTGGTAGATCTTCCTCCATCCTTT 280
III.2_1   CAGAGACTAGGATTGCAACCCCTGCCTTTTTTTGTTTTCCATTGGCTTGGTAGATCTTCCTCCATCCTTT 280
III.2_2   CAGAGACTAGGATTGCAACCCCTGCCTTTTTTTGTTTTCCATTGGCTTGGTAGATCTTCCTCCATCCTTT 280
          **********************************************************************

III.1_1   TATTTTGAGCCTATGTGTGTCTCTGCACGTGAGATAAAT----GGAATACAGCACACTGATGGGTCTTGA 350
III.2_1   TATTTTGAGCCTATGTGTGTCTCTGCACGTGAGATGGGTTTCCTGAATACAGCACACTGATGGGTCTTGA 350
III.2_2   TATTTTGAGCCTATGTGTGTCTCTGCACGTGAGATGGGTTTCCTGAATACAGCACACTGATGGGTCTTGA 350
          ***********************************   *     **************************

III.1_1   CTCTTTATCCAACTTGCCAGTCTGTGTCTTTTAATTGCAGAATTTAGTCCATTTATATTTAAAGTTAATA 420
III.2_1   CTCTTTATCCAACTTGCCAGTCTGTGTCTTTTAATTGCAGAATTTAGTCCATTTATATTTAAAGTTAATA 420
III.2_2   CTCTTTATCCAACTTGCCAGTCTGTGTCTTTTAATTGCAGAATTTAGTCCATTTATATTTAAAGTTAATA 420
          **********************************************************************

III.1_1   TTGTTATGTGTGAATTTGATCCTGTCATTATGATGTTAGCTGGTGATTTTGCTCATTAGTTGATGCAGTT 490
III.2_1   TTGTTATGTGTGAATTTGATCCTGTCATTATGATGTTAGCTGGTGATTTTGCTCATTAGTTGATGCAGTT 490
III.2_2   TTGTTATGTGTGAATTTGATCCTGTCATTATGATGTTAGCTGGTGATTTTGCTCATTAGTTGATGCAGTT 490
          **********************************************************************

III.1_1   TCTTCCTAGTCTCGATGGTCTTTACATTTTGGCATGATTTTGCAGCGGCTGGTACCGGTTGTTCCTTTCC 560
III.2_1   TCTTCCTAGTCTCGATGGTCTTTACATTTTGGCATGATTTTGCAGCGGCTGGTACCGGTTGTTCCTTTCC 560
III.2_2   TCTTCCTAGTCTCGATGGTCTTTACATTTTGGCATGATTTTGCAGCGGCTGGTACCGGTTGTTCCTTTCC 560
          **********************************************************************

III.1_1   ATGTTTAGCGCTTCCTTCAGGAGCTCTTTTAGGGCAGGCCTGGTGGTGACAAAATCTCTCAACATTTGCT 630
III.2_1   ATGTTTAGCGCTTCCTTCAGGAGCTCTTTTAGGGCAGGCCTGGTGGTGACAAAATCTCTCAACATTTGCT 630
III.2_2   ATGTTTAGCGCTTCCTTCAGGAGCTCTTTTAGGGCAGGCCTGGTGGTGACAAAATCTCTCAACATTTGCT 630
          **********************************************************************
```

```
III.1_1    TGTCTATAAAGTATTTTATTTCTCCTTCACTTATGAAGCTTAGTTTGGCTGGATATGAAATTCTGGGTTG 700
III.2_1    TGTCTATAAAGTATTTTATTTCTCCTTCACTTATGAAGCTTAGTTTGGCTGGATATGAAATTCTGGGTTG 700
III.2_2    TGTCTATAAAGTATTTTATTTCTCCTTCACTTATGAAGCTTAGTTTGGCTGGATATGAAATTCTGGGTTG 700
           **********************************************************************

III.1_1    AAAATTCTTTTCTTTAAGAATGTTGAATATTGGCCCCCACTCTCTTCTGGCTTGTAGGGTTTCTGCCGAG 770
III.2_1    AAAATTCTTTTCTTTAAGAATGTTGAATATTGGTCCCCACTCTCTTCTGGCTTGTAGGGTTTCTGCCGAG 770
III.2_2    AAAATTCTTTTCTTTAAGAATGTTGAATATTGGCCCCCACTCTCTTCTGGCTTGTAGGGTTTCTGCCGAG 770
           ********************************* ************************************

III.1_1    AGATCCGCTGTTAGTCTGATGGGCTTTC---TGAGGGTAACCTTCCCTT--TGCTTGGCTGCCCTTAACA 840
III.2_1    AGATCCGCTGTTAGTCTGATGGGCTTTCCTTTGAGGGTAACCCGACCTTTCTCTCTGGCTGCCCTTAACA 840
III.2_2    AGATCCGCTGTTAGTCTGATGGGCTTTCCTTTGAGGGTAACCCGACCTTTCTCTCTGGCTGCCCTTAACA 840
           ****************************   ***********   ****  *   ***************

III.1_1    TTTTTTCCTTCATTTCAACTTTGGTGAATCTGACAATTATGTGTCTTGGAGTTGCTCTTCTCGAGGAGTA 910
III.2_1    TTTTTTCCTTCATTTCAACTTTGGTGAATCTGACAATTATGTGTCTTGGAGTTGTTCTTCTCGAGGAGTA 910
III.2_2    TTTTTTCCTTCATTTCAACTTTGGTGAATCTGACAATTATGTGTCTTGGAGTTGCTCTTCTCGAGGAGTA 910
           ****************************************************** ***************

III.1_1    TCTTTGTGGCGTTCTCTGTATTTCCTGAATCTGAACGTTGGCCTGCCTTGCTAGATTGGGGAAGTTCTCC 980
III.2_1    TCTTTGTGGCGTTCTCTGTATTTCCTGAATCTGAACGTTGGCCTGCCTTGCTAGATTGGGGAAGTTCTCC 980
III.2_2    TCTTTGTGGCGTTCTCTGTATTTCCTGAATCTGAACGTTGGCCTGCCTTGCTAGATTGGGGAAGTTCTCC 980
           **********************************************************************

III.1_1    TGGATAATATCCTGCAGAGTGTTTTCCAACTTGGTTCCATTCTCCACATCACTTTCAGGTACACCAATCA 1050
III.2_1    TGGATAATATCCTGCAGAGTGTTTTCCAACTTGGTTCCATTCTCCACATCACTTTCAGGTACACCAATCA 1050
III.2_2    TGGATAATATCCTGCAGAGTGTTTTCCAACTTGGTTCCATTCTCCACATCACTTTCAGGTACACCAATCA 1050
           **********************************************************************

III.1_1    GACGTAGATTTGGTCTTTTCACATAGTCCCATATTTCTTGGAGGCTTTGCTCATTTCTTTTTATTCTTTT 1120
III.2_1    GACGTAGATTTGGTCTTTTCACATAGTCCCATATTTCTTGGAGGCTTTGCTCATTTCTTTTTATTCTTTT 1120
III.2_2    GACGTAGATTTGGTCTTTTCACATAGTCCCATATTTCTTGGAGGCTTTGCTCATTTCTTTTTATTCTTTT 1120
           **********************************************************************

III.1_1    TTCTCTAAACTTCCCTTCTCGCTTCATTTCATTCATTTCATCTTCCATTGCTGATACCCTTTCTTCCAGT 1190
III.2_1    TTCTCTAAACTTCCCTTCTCGCTTCATTTCATTCATTTCATCTTCCATTGCTGATACCCTTTCTTCCAGT 1190
III.2_2    TTCTCTAAACTTCCCTTCTCGCTTCATTTCATTCATTTCATCTTCCATTGCTGATACCCTTTCTTCCAGT 1190
           **********************************************************************

III.1_1    TG-----ATCGGCTCCTGAGGCTTGTGCATTCTTCACGTAGTTCTCGAGCCTTGGTTTTCAGCTCCATCA 1260
III.2_1    TGATCGCATCGGCTCCTGAGGCTTCTGCATTCTTCACGTAGTTCTCGAGCCTTGGTTTTCAGCTCCATCA 1260
III.2_2    TGATCGCATCGGCTCCTGAGGCTTCTGCATTCTTCACGTAGTTCTCGAGCCTTGGTTTTCAGCTCCATCA 1260
           **     ***************** *********************************************

III.1_1    GCTCCTTTAAGCACTTCTCTGTATTGGTTATTCTAGTTATACATTCTTCTAAATTTTTTTCAAAGTTTTC 1330
III.2_1    GCTCCTTTAAGCACTTCTCTGTATTGGTTATTCTAGTTATACATTCTTCTAAATTTTTTTCAAAGTTTTC 1330
III.2_2    GCTCCTTTAAGCACTTCTCTGTATTGGTTATTCTAGTTATACATTCTTCTAAATTTTTTTCAAAGTTTTC 1330
           **********************************************************************

III.1_1    AACTTCTTTGCCTTTGGTTTGAATGTCCTCCCGTAGCTCAGAGTAATTTGATCGTCTGAAGCCTTCTTCT 1400
III.2_1    AACTTCTTTGCCTTTGGTTTGAATGTCCTCCCGTAGCTCAGAGTAATTTGATCGTCTGAAGCCTTCTTCT 1400
III.2_2    AACTTCTTTGCCTTTGGTTTGAATGTCCTCCCGTAGCTCAGAGTAATTTGATCGTCTGAAGCCTTCTTCT 1400
           **********************************************************************

III.1_1    CTCAGCTCATCAAAATCATTCTCCATCCAGCTTTGTTCTGTTGCTGGTGAGGAACTGCGTTCCTTTGGAG 1470
III.2_1    CTCAGCTCATCAAAATCATTCTCCATCCAGCTTTGTTCTGTTGCTGGTGAGGAACTGCGTTCCTTTGGAG 1470
III.2_2    CTCAGCTCATCAAAATCATTCTCCATCCAGCTTTGTTCTGTTGCTGGTGAGGAACTGCGTTCCTTTGGAG 1470
           **********************************************************************

III.1_1    GAGGAGAGGCGCTCTGCGTTTTAGAGTTTCCAGTTTTTCTGTTCTGTTTTTTCCCCATCTTTGTGGTTTT 1540
III.2_1    GAGGAGAGGCGCTCTGCGTTTTAGAGTTTCCAGTTTTTCTGTTCTGTTTTTTCCCCATCTTTGTGGTTTT 1540
III.2_2    GAGGAGAGGCGCTCTGCGTTTTAGAGTTTCCAGTTTTTCTGTTCTGTTTTTTCCCCATCTTTGTGGTTTT 1540
           **********************************************************************

III.1_1    ATCTACTTTTGGTCTTTGATGATGGTGATGTACAGATGGGTTTTTGGTGTAGATGTCCTTTCTGGTTGTT 1610
III.2_1    ATCTACTTTTGGTCTTTGATGATGGTGATGTACAGATGGGTTTTCGGTGTAGATGTCCTTTCTGGTTGTT 1610
III.2_2    ATCTACTTTTGGTCTTTGATGATGGTGATGTACAGATGGGTTTTCGGTGTAGATGTCCTTTCTGGTTGTT 1610
           ******************************************** *************************

III.1_1    AGTTTTCCTTCTAACAGACAGGA-CTTTTAACACTCCACTGTCAACATTAGACAGATCAACGAGATAGAA 1680
III.2_1    AGTTTTCCTTCTAACAGACAGGACCCTTTAACACCCCACTGTCAACATTAGACAGATCAACGAGACAGAA 1680
III.2_2    AGTTTTCCTTCTAACAGACAGGACCCTTTAACACCCCACTGTCAACATTAGACAGATCAACGAGACAGAA 1680
           *********************** * ******** ****************************** ****
```

```
III.1_1     AGTCAACAAGGATACCCAGGAATTGAACTCAGCTCTGCACCAAGCAGACCTAATAGACATCTACAGAACT 1750
III.2_1     AGTCAACAAGGATACCCAGGAATTGAACTCAGCTCTGCACCAAGCAGACCTAATAGACATCTACAGAACT 1750
III.2_2     AGTCAACAAGGATACCCAGGAATTGAACTCAGCTCTGCACCAAGCAGACCTAATAGACATCTACAGAACT 1750
            **********************************************************************

III.1_1     CTCCACCCCAAATCAACAGAATATACATTTTTTTCAGCACCACACCACACCTATTCCAAAATTGACCACA 1820
III.2_1     CTCCACCCCAAATCAACAGAATATACATTTTTTTCAGCACCACACCACACCTATTCCAAAATTGACCACA 1820
III.2_2     CTCCACCCCAAATCAACAGAATATACATTTTTTTCAGCACCACACCACACCTATTCCAAAATTGACCACA 1820
            **********************************************************************

III.1_1     TAGTTGGAAGTAAAGCTCTCCTCAGCAAATGTAAAAGAACAGAAATTATAACAAACTATCTCTCAGACCA 1890
III.2_1     TAGTTGGAAGTAAAGCTCTCCTCAGCAAATGTAAAAGAACAGAAATTATAACAAACTATCTCTCAGACCA 1890
III.2_2     TAGTTGGAAGTAAAGCTCTCCTCAGCAAATGTAAAAGAACAGAAATTATAACAAACTATCTCTCAGACCA 1890
            **********************************************************************

III.1_1     CAGTGCAATCAAACTAGAACTCAGGATTAAGAATCTCACTCAAAGCCGCTCAACTACATGGAAACTGAAC 1960
III.2_1     CAGTGCAATCAAACTAGAACTCAGGATTAAGAATCTCACTCAAAGCCGCTCAACTACATGGAAACTGAAC 1960
III.2_2     CAGTGCAATCAAACTAGAACTCAGGATTAAGAATCTCACTCAAAGCCGCTCAACTACATGGAAACTGAAC 1960
            **********************************************************************

III.1_1     AACCTGCTCCTGAATGACTACTGGGTACATAACGAAATGAAGGCAGAAATAAAGATGTTCTTTGAAACCA 2030
III.2_1     AACCTGCTCCTGAATGACTACTGGGTACATAACGAAATGAAGGCAGAAATAAAGATGTTCTTTGAAACCA 2030
III.2_2     AACCTGCTCCTGAATGACTACTGGGTACATAACGAAATGAAGGCAGAAATAAAGATGTTCTTTGAAACCA 2030
            **********************************************************************

III.1_1     ACGAGAACAAAGACACCACATACCAGAATCTCTGGGACGCATTCAAAGCAGTGTGTAGAGGGAAATTTAT 2100
III.2_1     ACGAGAACAAAGACACCACATACCAGAATCTCTGGGACGCATTCAAAGCAGTGTGTAGAGGGAAATTTAT 2100
III.2_2     ACGAGAACAAAGACACCACATACCAGAATCTCTGGGACGCATTCAAAGCAGTGTGTAGAGGGAAATTTAT 2100
            **********************************************************************

III.1_1     AGCACTAAATGCCTAAGCAAGAGAAAGCAGGAAAGATCCAAAATTGACACCCTAACATCACAATTAAAAG 2170
III.2_1     AGCACTAAATGCCTA--AAAGAGAAAGCAGGAAAGATCCAAAATTGACACCCTAACATCACAATTAAAAG 2170
III.2_2     AGCACTAAATGCCTA--CAAGAGAAAGCAGGAAAGATCCAAAATTGACACCCTAACATCACAATTAAAAG 2170
            ***************   ****************************************************

III.1_1     AACTAGAAAAGCAAGAGCAAACACATTCAAAAGCTAGCAGAAGGCAAGAAATAACTAAAATCAGAGCAGA 2240
III.2_1     AACTAGAAAAGCAAGAGCAAACACATTCAAAAGCTAGCAGAAGGCAAGAAATAACTAAAATCAGAGCAGA 2240
III.2_2     AACTAGAAAAGCAAGAGCAAACACATTCAAAAGCTAGCAGAAGGCAAGAAATAACTAAAATCAGAGCAGA 2240
            **********************************************************************

III.1_1     ACTGAAGGAAATAGAGACACAAAAAACCCTTC-AAAAAATCAATGAATCCAGGAGCTGGTTTTTTGAAAG 2310
III.2_1     ACTGAAGGAAATAGAGACACAAAAAACCCTTCAAAAAAATCAATGAATCCAGGAGCTGGTTTTTTGAAAG 2310
III.2_2     ACTGAAGGAAATAGAGACACAAAAAACCCTTC-AAAAAATCAATGAATCCAGGAGCTGGTTTTTTGAAAG 2310
            ******************************** *************************************

III.1_1     GATCAA-AAAATTGATAGACCGCTAGCAAGACTAATAAAGAAAAAAAGAGAGAAGAATCAAATAGACACA 2380
III.2_1     GATCAACAAAATTGATAGACCGCTAGCAAGACTAATAAAGAAAAAAAGAGAGAAGAATCAAATAGACACA 2380
III.2_2     GATCAACAAAATTGATAGACCGCTAGCAAGACTAATAAAGAAAAAAAGAGAGAAGAATCAAATAGACACA 2380
            ****** ***************************************************************

III.1_1     ATAAAAAATGATAAAGGGGATATCACCACCGATCCCACAGAAATACAAACTACCATCAGAGAATACTACA 2450
III.2_1     ATAAAAAATGATAAAGGGGATATCACCACCGATCCCACAGAAATACAAACTACCATCAGAGAATACTACA 2450
III.2_2     ATAAAAAATGATAAAGGGGATATCACCACCGATCCCACAGAAATACAAACTACCATCAGAGAATACTACA 2450
            **********************************************************************

III.1_1     AACACCTCTACGCAAATAAACTAGAAAATCTAGAAGAAATGGATACATTCCTCGACACATACACTCTCCC 2520
III.2_1     AACACCTCTACGCAAATAAACTAGAAAATCTAGAAGAAATGGATACATTCCTCGACACATACACTCTCCC 2520
III.2_2     AACACCTCTACGCAAATAAACTAGAAAATCTAGAAGAAATGGATACATTCCTCGACACATACACTCTCCC 2520
            **********************************************************************

III.1_1     AAGACTAAACCAGGAAGAAGTTGAATCTCTGAATCGATCAATAACAGGCTCTGAAATTGTGGCAATAATC 2590
III.2_1     AAGACTAAACCAGGAAGAAGTTGAATCTCTGAATCGACCAATAACAGGCTCTGAAATTGTGGCAATAATC 2590
III.2_2     AAGACTAAACCAGGAAGAAGTTGAATCTCTGAATCGACCAATAACAGGCTCTGAAATTGTGGCAATAATC 2590
            ************************************* ********************************

III.1_1     AATAGTTTACCAACCAAAAAGAGTCCAGGACCAGATGGATTCACAGCCGAATTCTACCAGAGGTACAAGG 2660
III.2_1     AATAGTTTACCAACCAAAAAGAGTCCAGGACCAGATGGATTCACAGCCGAATTCTACCAGAGGTACAAGG 2660
III.2_2     AATAGTTTACCAACCAAAAAGAGTCCAGGACCAGATGGATTCACAGCCGAATTCTACCAGAGGTACAAGG 2660
            **********************************************************************

III.1_1     AGGAACTGGTACCATTCCTTCTGAAACTATTCCAATCAATAGAAAAAGAGGGAATCCTCCCTAACTCATT 2730
III.2_1     AGGAACTGGTACCATTCCTTCTGAAACTATTCCAATCAATAGAAAAAGAGGGAATCCTCCCTAACTCATT 2730
III.2_2     AGGAACTGGTACCATTCCTTCTGAAACTATTCCAATCAATAGAAAAAGAGGGAATCCTCCCTAACTCATT 2730
            **********************************************************************
```

```
III.1_1    TTATGAGGCCAGCATCATTCTGATACCAAAGCCGGGCAGAGACACAACCAAAAAAGAGAATTTTAGACCA 2800
III.2_1    TTATGAGGCCAGCATCATTCTGATACCAAAGCCGGGCAGAGACACAACCAAAAAAGAGAATTTTAGACCA 2800
III.2_2    TTATGAGGCCAGCATCATTCTGATACCAAAGCCGGGCAGAGACACAACCAAAAAAGAGAATTTTAGACCA 2800
           **********************************************************************

III.1_1    ATATCCTTGATGAACATTGATGCAAAAATCCTCAATAAAATACTGGCAAACCGAATC---CAGCACATCA 2870
III.2_1    ATATCCTTGATGAACATTGATGCAAAAATCCTCAATAAAATACTGGCAAACCGAATCCAGCAGCACATCA 2870
III.2_2    ATATCCTTGATGAACATTGATGCAAAAATCCTCAATAAAATACTGGCAAACCGAATCCAGCAGCACATCA 2870
           *********************************************************   **********

III.1_1    AAAAGCTTATCCACCATGATCAAGTGGGCTTCAT-CCCTGGGATGCAAGGCTGGTTCAATATACGCAAAT 2940
III.2_1    AAAAGCTTATCCACCATGATCAAGTGGGCTTCATCCCCTGGGATGCAAGGCTGGTTCAATATACGCAAAT 2940
III.2_2    AAAAGCTTATCCACCATGATCAAGTGGGCTTCAT-CCCTGGGATGCAAGGCTGGTTCAATATACGCAAAT 2940
           ********************************** ***********************************

III.1_1    CAATAAATGTAATCCAGCATATAAACAGAGCCAAAGACAAAAACCACATGATTATCTCAATAGATGCAGA 3010
III.2_1    C----AATGTAATCCAGCATATAAACAGAGCCAAAGACAAAAACCACATGATTATCTCAATAGATGCAGA 3010
III.2_2    CAATAAATGTAATCCAGCATATAAACAGAGCCAAAGACAAAAACCACATGATTATCTCAATAGATGCAG- 3010
           *    ****************************************************************

III.1_1    AAAAGCCTTTGACAAAATTCAACAACCCTTCATGCTAAAAACTCTCAATAAATTAGGTATTGATGGGACG 3080
III.2_1    AAAAGCCTTTGACAAAATTCAACAACCCTTCATGCTAAAAACTCTCAATAAATTAGGTATTGATGGGACG 3080
III.2_2    AAAAGCCTTTGACAAAATTCAACAACCCTTCATGCTAAAAACTCTCAATAAATTAGGTATTGATGGGACG 3080
           **********************************************************************

III.1_1    TATTTCAAAATAATAAGAGCTATCTATGACAAACCCACAGCCAATATCATACTGAATGGGCAAAAACTGG 3150
III.2_1    TATTTCAAAATAATAAGAGCTATCTATGACAAACCCACAGCCAATATCATACTGAATGGGCAAAAACTGG 3150
III.2_2    TACTTCAAAATAATAAGAGCTATCTATGACAAACCCACAGCCAATATCATACTGAATGGGCAAAAACTGG 3150
           ** *******************************************************************

III.1_1    AAGCATTCCCTTTGAAAACCGGCACAAGACAGGGATGCCCTCTCTCACCGCTCCTATTCAACATAGTGTT 3220
III.2_1    AAGCATTCCCTTTGAAAACCGGCACAAGACAGGGATGCCCTCTCTCACCGCTCCTATTCAACATAGTGTT 3220
III.2_2    AAGCATTCCCTTTGAAAACCGGCACAAGACAGGGATGCCCTCTCTCACCGCTCCTATTCAACATAGTGTT 3220
           **********************************************************************

III.1_1    GGAAGTTCTGGCCAGGGCAATCAGGCAGGAGAAGGAAATAAAGGGTATTCAATTAGGAAAAGAGGAAGTC 3290
III.2_1    GGAAGTTCTGGCCAGGGCAATCAGGCAGGAGAAGGAAATAAAGGGTATTCAATTAGGAAAAGAGGAAGTC 3290
III.2_2    GGAAGTTCTGGCCAGGGCAATCAGGCAGGAGAAGGAAATAAAGGGTATTCAATTAGGAAAAGAGGAAGTC 3290
           **********************************************************************

III.1_1    AAATTGTCCCTGTTTGCAGACGACATGATTGTTTATCTAGAAAACCCCATCGTCTCAGCCCAAAATCTCC 3360
III.2_1    AAATTGTCCCTGTTTGCAGACGACATGATTGTTTATCTAGAAAACCCCATCGTCTCAGCCCAAAATCTCC 3360
III.2_2    AAATTGTCCCTGTTTGCAGACGACATGATTGTTTATCTAGAAAACCCCATCGTCTCAGCCCAAAATCTCC 3360
           **********************************************************************

III.1_1    TTAAGCTGATAAGCAACTTCAGCAAAGTCTCAGGATACAAAATCAATGTACAAAAATCACAAGCATTCTT 3430
III.2_1    TTAAGCTGATAAGCAACTTCAGCAAAGTCTCAGGATACAAAATCAATGTACAAAAATCACAAGCATTCTT 3430
III.2_2    TTAAGCTGATAAGCAACTTCAGCAAAGTCTCAGGATACAAAATCAATGTACAAAAATCACAAGCATTCTT 3430
           **********************************************************************

III.1_1    ATACACCAACAACAGACAAACAGAGAGCCAAATCATGGGTGAACTCCCATTCACAATTGCTTCAAAGAGA 3500
III.2_1    ATACACCAACAACAGACAAACAGAGAGCCAAATCATGAGTGAACTCCCATTCACAATTGCTTCAAAGAGA 3500
III.2_2    ATACACCAACAACAGACAAACAGAGAGCCAAATCATGGGTGAACTCCCATTCACAATTGCTTCAAAGAGA 3500
           ************************************* ********************************

III.1_1    ATAAAATACCTAGGAATCCAACTTACAAGGGATGTGAAGGACCTCTTCAAGGAGAACTACAAACCACTGC 3570
III.2_1    ATAAAATACCTAGGAATCCAACTTACAAGGGATGTGAAGGACCTCTTCAAGGAGAACTACAAACCACTGC 3570
III.2_2    ATAAAATACCTAGGAATCCAACTTACAAGGGATGTGAAGGACCTCTTCAAGGAGAACTACAAACCACTGC 3570
           **********************************************************************

III.1_1    TCAAGGAAATAAAAGAGGAGACAAACAAATGGAAGAACATTCCATGCTCATGGGTAGGAAGAATCAATAT 3640
III.2_1    TCAAGGAAATAAAAGAGGAGACAAACAAATGGAAGAACATTCCATGCTCATGGGTAGGAAGAATCAATAT 3640
III.2_2    TCAAGGAAATAAAAGAGGAGACAAACAAATGGAAGAACATTCCATGCTCATGGGTAGGAAGAATCAATAT 3640
           **********************************************************************

III.1_1    CGTGAAAATGGCCATACTGCCCAAGGTAATTTACAGATTCAATGCCATCCCCATCAAGCTACCAATGACT 3710
III.2_1    CGTGAAAATGGCCATACTGCCCAAGGTAATTTACAGATTCAATGCCATCCCCATCAAGCTACCAATGACT 3710
III.2_2    CGTGAAAATGGCCATACTGCCCAAGGTAATTTACAGATTCAATGCCATCCCCATCAAGCTACCAATGACT 3710
           **********************************************************************

III.1_1    TTCTTCACAGAATTGGAAAAAACTACTTTAAAGTTCATATGGAACCAAAAAAGAGCCCGCATTGCCAAGT 3780
III.2_1    TTCTTCACAGAATTGGAAAAAACTACTTTAAAGTTCATATGGAACCAAAAAAGAGCCCGCATTGCCAAGT 3780
III.2_2    TTCTTCACAGAATTGGAAAAAACTACTTTAAAGTTCATATGGAACCAAAAAAGAGCCCGCATTGCCAAGT 3780
           **********************************************************************
```

```
III.1_1   CAATCCTAAGCCAAAAGAACAAAGCTGGAGGCATCACACTACCTGACTTCAAACTATACTACAAGGCTAC 3850
III.2_1   CAATCCTAAGCCAAAAGAACAAAGCTGGAGGCATCACACTACCTGACTTCAAACTATACTACAAGGCTAC 3850
III.2_2   CAATCCTAAGCCAAAAGAACAAAGCTGGAGGCATCACACTACCTGACTTCAAACTATACTACAAGGCTAC 3850
          **********************************************************************

III.1_1   AGTAACCAAAACAGCATGGTACTGGTACCAAAACAGAGATATAGATCAATGGAACAGAACAGAGCCCTCA 3920
III.2_1   AGTAACCAAAACAGCATGGTACTGGTACCAAAACAGAGATATAGATCAATGGAACAGAACAGAGCCCTCA 3920
III.2_2   AGTAACCAAAACAGCATGGTACTGGTACCAAAACAGAGATATAGATCAATGGAACAGAACAGAGCCCTCA 3920
          **********************************************************************

III.1_1   GAAATAATGCCGCATATCTACAACTATCTGATCTTTGACAAACCTGAGAAAAACAAGCAATGGGGAAAGG 3990
III.2_1   GAAATAATGCCGCATATCTACAACTATCTGATCTTTGACAAACCTGAGAAAAACAAGCAATGGGGAAAGG 3990
III.2_2   GAAATAATGCCGCATATCTACAACTATCTGATCTTTGACAAACCTGAGAAAAACAAGCAATGGGGAAAGG 3990
          **********************************************************************

III.1_1   ATTCCCTATTTAATAAATGGTGCTGGGAAAACTGGCTAGCCATATGTAGAAAGCTGAAACTGGATCCCTT 4060
III.2_1   ATTCCCTATTTAATAAATGGTGCTGGGAAAACTGGCTAGCCATATGTAGAAAGCTGAAACTGGATCCCTT 4060
III.2_2   ATTCCCTATTTAATAAATGGTGCTGGGAAAACTGGCTAGCCATATGTAGAAAGCTGAAACTGGATCCCTT 4060
          **********************************************************************

III.1_1   CCTTACACCTTATACAAAAATCAATTCAAGATGGATTAAAGATTTAAACGTTAAACTCAAAACCATAAAA 4130
III.2_1   CCTTACACCTTATACAAAAATCAATTCAAGATGGATTAAAGATTTAAACGTTAAACCTAAAACCATAAAA 4130
III.2_2   CCTTACACCTTATACAAAAATCAATTCAAGATGGATTAAAGATTTAAACGTTAAACCTAAAACCATAAAA 4130
          *********************************************************  ***********

III.1_1   ACCCTAGAAGAAAACCTAGGCATTACCATTCAGGACATAGGCGTGGGCAAGGACTTCATGTCCAAAACAC 4200
III.2_1   ACCCTAGAAGAAAACCTAGGCATTACCATTCAGGACATAGGCGTGGGCAAGGACTTCATGTCCAAAACAC 4200
III.2_2   ACCCTAGAAGAAAACCTAGGCATTACCATTCAGGACATAGGCGTGGGCAAGGACTTCATGTCCAAAACAC 4200
          **********************************************************************

III.1_1   CAAAAGCAATGGCAACAAAAGACAAAATTGACAAATGGGATCTAATTAAACTAAAGAGCTTCTGCACAGC 4270
III.2_1   CAAAAGCAATGGCAACAAAAGACAAAATTGACAAATGGGATCTAATTAAACTAAAGAGCTTCTGCACAGC 4270
III.2_2   CAAAAGCAATGGCAACAAAAGACAAAATTGACAAATGGGATCTAATTAAACTAAAGAGCTTCTGCACAGC 4270
          **********************************************************************

III.1_1   AAAAGAAACTACCATCAGAGTGAACAGGCAACCTACAACATGGGAGAAAATTTTTGCAACCTACTCATCT 4340
III.2_1   AAAAGAAACTACCATCAGAGTGAACAGGCAACCTACAACATGGGAGAAAATTTTTGCAACCTACTCATCT 4340
III.2_2   AAAAGAAACTACCATCAGAGTGAACAGGCAACCTACAACATGGGAGAAAATTTTTGCAACCTACTCATCT 4340
          **********************************************************************

III.1_1   GACAAAGGGCTAATATCCAGAATCTACAATGAACTCAAACAAATTTACAAGAAAAAAACAAACAACCCCA 4410
III.2_1   GACAAAGGGCTAATATCCAGAATCTACAATGAACTCAAACAAATTTACAAGAAAAAAACAAACAACCCCA 4410
III.2_2   GACAAAGGGCTAATATCCAGAATCTACAATGAACTCAAACAAATTTACAAGAAAAAAACAAACAACCCCA 4410
          **********************************************************************

III.1_1   TCAAAAAGTGGGCGAAGGACATGAACAGACACTTCTCAAAAGAAGACATTTATGCAGCCAAAAAACACAT 4480
III.2_1   TCAAAAAGTGGGCGAAGGACATGAACAGACACTTCTCAAAAGAAGACATTTATGCAGCCAAAAAACACAT 4480
III.2_2   TCAAAAAGTGGGCGAAGGACATGAACAGACACTTCTCAAAAGAAGACATTTATGCAGCCAAAAAACACAT 4480
          **********************************************************************

III.1_1   GAAGAAATGCTCATCATCACTGGCCATCAGAGAAATGCAAATCAAAACCACTATGAGATATCATCTCACA 4550
III.2_1   GAAGAAATGCTCATCATCACTGGCCATCAGAGAAATGCAAATCAAAACCACTATGAGATATCATCTCACA 4550
III.2_2   GAAGAAATGCTCATCATCACTGGCCATCAGAGAAATGCAAATCAAAACCACTATGAGATATCATCTCACA 4550
          **********************************************************************

III.1_1   CCAGTTAGAATGGCAATCATTAAAAAGTCAGGAAACAACAGGTGCTGGAGAGGATGCGGAGAAATAGGAA 4620
III.2_1   CCAGTTAGAATGGCAATCATTAAAAAGTCAGGAAACAACAGGTGCTGGAGAGGATGCGGAGAAATAGGAA 4620
III.2_2   CCAGTTAGAATGGCAATCATTAAAAAGTCAGGAAACAACAGGTGCTGGAGAGGATGCGGAGAAATAGGAA 4620
          **********************************************************************

III.1_1   CACTTTTACACTGTTGGTGGGACTGTAAACTAGTTCAACCATTGTGGAAGTCAGTGTGGCGATTCCTCAG 4690
III.2_1   CACTTTTACACTGTTGGTGGGACTGTAAACTAGTTCAACCATTGTGGAAGTCAGTGTGGCGATTCCTCAG 4690
III.2_2   CACTTTTACACTGTTGGTGGGACTGTAAACTAGTTCAACCATTGTGGAAGTCAGTGTGGCGATTCCTCAG 4690
          **********************************************************************

III.1_1   GGATCTAGAACTAGAAATACCATTTGACCCAGCCATCCCATTACTGGGTATATACCCAAATGAGTATAAA 4760
III.2_1   GGATCTAGAACTAGAAATACCATTTGACCCAGCCATCCCATTACTGGGTATATACCCAAATGAGTATAAA 4760
III.2_2   GGATCTAGAACTAGAAATACCATTTGACCCAGCCATCCCATTACTGGGTATATACCCAAATGAGTATAAA 4760
          **********************************************************************

III.1_1   TCATGCTGCTATAAAGACACATGCACACGTATGTTTATTGCGGCACTATTCACAATAGCAAAGACTTGGA 4830
III.2_1   TCATGCTGCTATAAAGACACATGCACACGTATGTTTATTGCGGCACTATTCACAATAGCAAAGACTTGGA 4830
III.2_2   TCATGCTGCTATAAAGACACATGCACACGTATGTTTATTGCGGCACTATTCACAATAGCAAAGACTTGGA 4830
          **********************************************************************
```

```
III.1_1   ACCAACCCAAATGTCCAACAATGATAGACTGGATTAAGAAAATGTGGCACATATACACCATGGAATACTA 4900
III.2_1   ACCAACCCAAATGTCCAACAATGATAGACTGGATTAAGAAAATGTGGCACATATACACCATGGAATACTA 4900
III.2_2   ACCAACCCAAATGTCCAACAATGATAGACTGGATTAAGAAAATGTGGCACATATACACCATGGAATACTA 4900
          **********************************************************************

III.1_1   TGCAGCCATAAAAAATGATGAGTTCATATCCTTTGTAGGGACATGGATG-AAATTGGAAACCATCATTCT 4970
III.2_1   TGCAGCCATAAAAAATGATGAGTTCATATCCTTTGTAGGGACATGGATGAAAATTGGAAATCATCATTCT 4970
III.2_2   TGCAGCCATAAAAAATGATGAGTTCATATCCTTTGTAGGGACATGGATG-AAATTGGAAACCATCATTCT 4970
          ************************************************* ********** *********

III.1_1   CAGCAAACTATCGCAAGAACAAAAAACCAAACACCGCATATTCTCACTCATAGGTGGGAATTGAACAATG 5040
III.2_1   CAGTAAACTATCGCAAGAACAAAAAACCAAACACCGCATATTCTCACTCATAGGTGGGAATTGAACAATG 5040
III.2_2   CAGTAAACTATCGCAAGAACAAAAAACCAAACACCGCATATTCTCACTCATAGGTGGGAATTGAACAATG 5040
          *** ******************************************************************

III.1_1   AGATCACATGGACCCAGGAAGGGGAACATCACACTCTGGGGACT-GTGGT-GGGCCGGGGGAGGGGGGAG 5110
III.2_1   AGATCACATGGACCCAGGAAGGGGAATATCACACTCTGGGGACT-GTGGTGGGGTC-GGGGAGGGGGGAG 5110
III.2_2   AGAT--CATGGACCCAGGAAGGGGAATATCACACTCTGGGGACTGGGGGTGGGGTCGGGGGAGGGGGGAG 5110
          ****  ******************** ***************** * *** *** * *************

III.1_1   GGATAGCATTGGGAGATATACCTAATGCTAGATGACACATTAGTGGGTGCAGCGCACCAGCATGGCACAT 5180
III.2_1   GGATAGCATTGGGAGATATACCTAATGCTAGATGACACATTAGTGGGTGCAGCGCACCAGCATGGCACAT 5180
III.2_2   GGATAGCATTGGGAGATATACCTAATGCTAGATGACACATTAGTGGGTGCAGCGCACCAGCATGGCACAT 5180
          **********************************************************************

III.1_1   GTATACATATGTAACTAACCTGCACAATGTGCACATGTACCCTAAAACTTAGAGTATAAT---AAAAAAA 5250
III.2_1   GTATACATATGTAACTAACCTGCACAATGTGCACATGTACCCTAAAACTTAGAGTATAAT--AAAAAAAA 5250
III.2_2   GTATACATATGTAACTAACCTGCACAATGTGCACATGTACCCTAAAACTTAGAGTATAATAAAAAAAAAA 5250
          ************************************************************   *******

III.1_1   AAAAAAAAAAAATAGAAAGAAAGAAATAAAT--AAAAAAA-GAAAAGAAAGAAAGAAAGAAAGAAAGAAA 5320
III.2_1   AAAAAAAAAAAATAGAAAGAAAGAAATAAAT-AAAAAGAAAGAAA-GAAAGAAAGAAAGAAAGAAAGAAA 5320
III.2_2   AAAAAAAAAAAATAGAAAGAAAGAAATAAATAAAAAAGAAAGAAA-GAAAGAAAGAAAGAAAGAAAGAAA 5320
          *******************************  **** ** **** ************************

III.1_1   GAAAGAAAGAAAGAAAGAAAGAAAGAAAGAAAGAAAGAAAGAAAGAAAGAAAGAAAGAGAAAAGCAAAAT 5390
III.2_1   GAAAGAAAGAAAGAAAGAAAGAAAGAAAGAAAGAAAGAAAGAAAGAAAGAAAGAAAGAGAAAAGCAAAAT 5390
III.2_2   GAAAGAAAGAAAG-AAGAAAGAAAGAAAGAAAGAAAGAAAGAAAGAAAGAAAGAAAGAGAAAAG--AAAT 5390
          ************* **************************************************  ****

III.1_1   GTCTATTCCGGCTCATTGCCTATTTTTT-GAATTTGGATTTT-GTTTTCTTGCTATTGAGTTGTTTGAGT 5460
III.2_1   GTCTATTCCGGTCTATTGCCTATTTTTT-GAATTTGGATTTTGGTTTTCTTGCTATTGAGTTGTTTGAGT 5460
III.2_2   GTCTATT-CAGTCTATTGCCTATTTTTTGGAATTTGGATTTTGGTTTTCTTGCTATTGAGTTGTTTGAGT 5460
          ******* * *   ************** ************* ***************************

III.1_1   TCTTTATATATTTTGAGTAATAATCCCTTATCAGATGTATGCTTTGCAAATAAA-TTTTCCATTCCATGC 5530
III.2_1   TCCTTATATATTTTGAGTAATAACTCCTTATCAGATGTATGCTTTGCAAATAAA-TTTCCCATTCCATGC 5530
III.2_2   TCCTTATATATTTTGAGTAATAACTCCTTATCAGATGTATGCTTTGCAAATAAATTTTCCCATTCCATGC 5530
          ** ********************  ***************************** *** ***********

III.1_1   -------AAAAAAAAAAAAAAAAAAAAAAAAAAAAAAAAAAAAAAAAAGAATGAGAGAATA 5590
III.2_1   -------------AAAAAAAAAAAAAAAAAAAAAAAAAAAAAAAAAAAGAATGAGAGAATA 5590
III.2_2   AAAAAAAAAAAAAAAAAAAAAAAAAAAAAAAAAAAAAAAAAAAAAAAGAATGAGAGAATA 5590
                       ***********************************************
```

The multiple sequence alignment was used construct a consensus sequence of the LINE-1 insertion in RP1 exon 4 of patients III.1 and III.2.

```
>RP1_exon4_LINE1_consensus_sequence
GTggGGTGTTAAAGTCTCCCATTATTAAtgtgTGGGAGTCTAAGTCTCTTTGTAGGTCACTGAGGACTTGC
TTTATGAATCTGGGTGCTCCTGTATTGGGTGCATAAATATTTAGGATAGTTAGCTCCTCTTGTTGAATTGA
TCCCTTTACCATTATGTAATGGCCTTCTTTGTCTCTTTTGATCTTTGTTGGTTTAAAGTCTGTTTTATCAG
AGACTAGGATTGCAACCCCTGCCTTTTTTTGTTTTCCATTGGCTTGGTAGATCTTCCTCCATCCTTTTATT
TTGAGCCTATGTGTGTCTCTGCACGTGAGATgggTttcctGAATACAGCACACTGATGGGTCTTGACTCTT
TATCCAACTTGCCAGTCTGTGTCTTTTAATTGCAGAATTTAGTCCATTTATATTTAAAGTTAATATTGTTA
TGTGTGAATTTGATCCTGTCATTATGATGTTAGCTGGTGATTTTGCTCATTAGTTGATGCAGTTTCTTCCT
AGTCTCGATGGTCTTTACATTTTGGCATGATTTTGCAGCGGCTGGTACCGGTTGTTCCTTTCCATGTTTAG
CGCTTCCTTCAGGAGCTCTTTTAGGGCAGGCCTGGTGGTGACAAAATCTCTCAACATTTGCTTGTCTATAA
AGTATTTTATTTCTCCTTCACTTATGAAGCTTAGTTTGGCTGGATATGAAATTCTGGGTTGAAAATTCTTT
```

TCTTTAAGAATGTTGAATATTGGcCCCCACTCTCTTCTGGCTTGTAGGGTTTCTGCCGAGAGATCCGCTGT
TAGTCTGATGGGCTTTCcttTGAGGGTAACCcgaCCTTtcTctcTGGCTGCCCTTAACATTTTTTCCTTCA
TTTCAACTTTGGTGAATCTGACAATTATGTGTCTTGGAGTTGcTCTTCTCGAGGAGTATCTTTGTGGCGTT
CTCTGTATTTCCTGAATCTGAACGTTGGCCTGCCTTGCTAGATTGGGGAAGTTCTCCTGGATAATATCCTG
CAGAGTGTTTTCCAACTTGGTTCCATTCTCCACATCACTTTCAGGTACACCAATCAGACGTAGATTTGGTC
TTTTCACATAGTCCCATATTTCTTGGAGGCTTTGCTCATTTCTTTTTATTCTTTTTTCTCTAAACTTCCCT
TCTCGCTTCATTTCATTCATTTCATCTTCCATTGCTGATACCCTTTCTTCCAGTTGatcgcATCGGCTCCT
GAGGCTTcTGCATTCTTCACGTAGTTCTCGAGCCTTGGTTTTCAGCTCCATCAGCTCCTTTAAGCACTTCT
CTGTATTGGTTATTCTAGTTATACATTCTTCTAAATTTTTTTCAAAGTTTTCAACTTCTTTGCCTTTGGTT
TGAATGTCCTCCCGTAGCTCAGAGTAATTTGATCGTCTGAAGCCTTCTTCTCTCAGCTCATCAAAATCATT
CTCCATCCAGCTTTGTTCTGTTGCTGGTGAGGAACTGCGTTCCTTTGGAGGAGGAGAGGCGCTCTGCGTTT
TAGAGTTTCCAGTTTTTCTGTTCTGTTTTTTCCCCATCTTTGTGGTTTTATCTACTTTTGGTCTTTGATGA
TGGTGATGTACAGATGGGTTTTcGGTGTAGATGTCCTTTCTGGTTGTTAGTTTTCCTTCTAACAGACAGGA
cCcTTTAACACcCCACTGTCAACATTAGACAGATCAACGAGAcAGAAAGTCAACAAGGATACCCAGGAATT
GAACTCAGCTCTGCACCAAGCAGACCTAATAGACATCTACAGAACTCTCCACCCCAAATCAACAGAATATA
CATTTTTTTCAGCACCACACCACACCTATTCCAAAATTGACCACATAGTTGGAAGTAAAGCTCTCCTCAGC
AAATGTAAAAGAACAGAAATTATAACAAACTATCTCTCAGACCACAGTGCAATCAAACTAGAACTCAGGAT
TAAGAATCTCACTCAAAGCCGCTCAACTACATGGAAACTGAACAACCTGCTCCTGAATGACTACTGGGTAC
ATAACGAAATGAAGGCAGAAATAAAGATGTTCTTTGAAACCAACGAGAACAAAGACACCACATACCAGAAT
CTCTGGGACGCATTCAAAGCAGTGTGTAGAGGGAAATTTATAGCACTAAATGCCTAcAAGAGAAAGCAGGA
AAGATCCAAAATTGACACCCTAACATCACAATTAAAAGAACTAGAAAAGCAAGAGCAAACACATTCAAAAG
CTAGCAGAAGGCAAGAAATAACTAAAATCAGAGCAGAACTGAAGGAAATAGAGACACAAAAAACCCTTCAA
AAAATCAATGAATCCAGGAGCTGGTTTTTTGAAAGGATCAAcAAAATTGATAGACCGCTAGCAAGACTAAT
AAAGAAAAAAAGAGAGAAGAATCAAATAGACACAATAAAAAATGATAAAGGGGATATCACCACCGATCCCA
CAGAAATACAAACTACCATCAGAGAATACTACAAACACCTCTACGCAAATAAACTAGAAAATCTAGAAGAA
ATGGATACATTCCTCGACACATACACTCTCCCAAGACTAAACCAGGAAGAAGTTGAATCTCTGAATCGAcC
AATAACAGGCTCTGAAATTGTGGCAATAATCAATAGTTTACCAACCAAAAAGAGTCCAGGACCAGATGGAT
TCACAGCCGAATTCTACCAGAGGTACAAGGAGGAACTGGTACCATTCCTTCTGAAACTATTCCAATCAATA
GAAAAAGAGGGAATCCTCCCTAACTCATTTTATGAGGCCAGCATCATTCTGATACCAAAGCCGGGCAGAGA
CACAACCAAAAAAGAGAATTTTAGACCAATATCCTTGATGAACATTGATGCAAAAATCCTCAATAAAATAC
TGGCAAACCGAATCcagCAGCACATCAAAAAGCTTATCCACCATGATCAAGTGGGCTTCATCCCTGGGATG
CAAGGCTGGTTCAATATACGCAAATCaataAATGTAATCCAGCATATAAACAGAGCCAAAGACAAAAACCA
CATGATTATCTCAATAGATGCAGaAAAAGCCTTTGACAAAATTCAACAACCCTTCATGCTAAAAACTCTCA
ATAAATTAGGTATTGATGGGACGTAtTTCAAAATAATAAGAGCTATCTATGACAAACCCACAGCCAATATC
ATACTGAATGGGCAAAAACTGGAAGCATTCCCTTTGAAAACCGGCACAAGACAGGGATGCCCTCTCTCACC
GCTCCTATTCAACATAGTGTTGGAAGTTCTGGCCAGGGCAATCAGGCAGGAGAAGGAAATAAAGGGTATTC
AATTAGGAAAAGAGGAAGTCAAATTGTCCCTGTTTGCAGACGACATGATTGTTTATCTAGAAAACCCCATC
GTCTCAGCCCAAAATCTCCTTAAGCTGATAAGCAACTTCAGCAAAGTCTCAGGATACAAAATCAATGTACA
AAAATCACAAGCATTCTTATACACCAACAACAGACAAACAGAGAGCCAAATCATGgGTGAACTCCCATTCA
CAATTGCTTCAAAGAGAATAAAATACCTAGGAATCCAACTTACAAGGGATGTGAAGGACCTCTTCAAGGAG
AACTACAAACCACTGCTCAAGGAAATAAAAGAGGAGACAAACAAATGGAAGAACATTCCATGCTCATGGGT
AGGAAGAATCAATATCGTGAAAATGGCCATACTGCCCAAGGTAATTTACAGATTCAATGCCATCCCCATCA
AGCTACCAATGACTTTCTTCACAGAATTGGAAAAAACTACTTTAAAGTTCATATGGAACCAAAAAAGAGCC
CGCATTGCCAAGTCAATCCTAAGCCAAAAGAACAAAGCTGGAGGCATCACACTACCTGACTTCAAACTATA
CTACAAGGCTACAGTAACCAAAACAGCATGGTACTGGTACCAAAACAGAGATATAGATCAATGGAACAGAA
CAGAGCCCTCAGAAATAATGCCGCATATCTACAACTATCTGATCTTTGACAAACCTGAGAAAAACAAGCAA
TGGGGAAAGGATTCCCTATTTAATAAATGGTGCTGGGAAAACTGGCTAGCCATATGTAGAAAGCTGAAACT
GGATCCCTTCCTTACACCTTATACAAAAATCAATTCAAGATGGATTAAAGATTTAAACGTTAAACctAAAA
CCATAAAAACCCTAGAAGAAAACCTAGGCATTACCATTCAGGACATAGGCGTGGGCAAGGACTTCATGTCC
AAAACACCAAAAGCAATGGCAACAAAAGACAAAATTGACAAATGGGATCTAATTAAACTAAAGAGCTTCTG
CACAGCAAAAGAAACTACCATCAGAGTGAACAGGCAACCTACAACATGGGAGAAAATTTTTGCAACCTACT
CATCTGACAAAGGGCTAATATCCAGAATCTACAATGAACTCAAACAAATTTACAAGAAAAAAACAAACAAC
CCCATCAAAAAGTGGGCGAAGGACATGAACAGACACTTCTCAAAAGAAGACATTTATGCAGCCAAAAAACA
CATGAAGAAATGCTCATCATCACTGGCCATCAGAGAAATGCAAATCAAAACCACTATGAGATATCATCTCA
CACCAGTTAGAATGGCAATCATTAAAAAGTCAGGAAACAACAGGTGCTGGAGAGGATGCGGAGAAATAGGA
ACACTTTTACACTGTTGGTGGGACTGTAAACTAGTTCAACCATTGTGGAAGTCAGTGTGGCGATTCCTCAG
GGATCTAGAACTAGAAATACCATTTGACCCAGCCATCCCATTACTGGGTATATACCCAAATGAGTATAAAT
CATGCTGCTATAAAGACACATGCACACGTATGTTTATTGCGGCACTATTCACAATAGCAAAGACTTGGAAC

CAACCCAAATGTCCAACAATGATAGACTGGATTAAGAAAATGTGGCACATATACACCATGGAATACTATGC
AGCCATAAAAAATGATGAGTTCATATCCTTTGTAGGGACATGGATGAAATTGGAAAcCATCATTCTCAGtA
AACTATCGCAAGAACAAAAAACCAAACACCGCATATTCTCACTCATAGGTGGGAATTGAACAATGAGATca
CATGGACCCAGGAAGGGGAAtATCACACTCTGGGGACTGtGGTgGGGtCgGGGGAGGGGGGAGGGATAGCA
TTGGGAGATATACCTAATGCTAGATGACACATTAGTGGGTGCAGCGCACCAGCATGGCACATGTATACATA
TGTAACTAACCTGCACAATGTGCACATGTACCCTAAAACTTAGAGTATAATaAAAAAAAAAAAAAAAAAAA
TAGAAAGAAAGAAATAAATaAAAAgAAaGAAAGAAAGAAAGAAAGAAAGAAAGAAAGAAAGAAAGAAAGaA
AGAAAGAAAGAAAGAAAGAAAGAAAGAAAGAAAGAAAGAAAGAGAAAAGcaAAATGTCTATTcCgGtctAT
TGCCTATTTTTTGAATTTGGATTTTgGTTTTCTTGCTATTGAGTTGTTTGAGTTCcTTATATATTTTGAGT
AATAActCCTTATCAGATGTATGCTTTGCAAATAAATTTcCCATTCCATGCaaaaaaAAAAAAAAAAAAAA
AAAAAAAAAAAAAAAAAAAAGAATGAGAGAATA
