# Supplementary material for: Characterisation of a LINE-1 Insertion in the RP1 Gene by Targeted Adaptive Nanopore Sequencing in a Family with Retinitis Pigmentosa
Source: Hum Mutat. 2024 Feb 9;2024:6580561. doi: 10.1155/2024/6580561 (PMC11919084; doi:10.1155/2024/6580561)
Supplement: Supplementary 3 — Table S1: genetic positions and overlapping genes included in the custom target panel for targeted adaptive nanopore sequencing. [file 6580561.f3.pdf]

Supplementary Table S1. Genetic positions and overlapping genes included in the custom target panel for targeted adaptive nanopore sequencing

Chromosome	Start position	End position	Genes overlapping with the position
chr1	908923	959575	LINC02593;SAMD11;NOC2L
chr1	1497162	1549685	ATAD3B;ATAD3A;TMEM240
chr1	2388964	2428797	MORN1;RER1;PEX10
chr1	5847811	6007473	MIR4689;NPHP4
chr1	6409776	6476367	HES2;MIR4252;ESPN;TNFRSF25
chr1	8337397	8863921	SLC45A1;RERE;RERE-AS1;SNORD128
chr1	9928428	10000501	LZIC;TMEM274P;MIR5697;NMNAT1
chr1	10457288	10645758	DFFA;PEX14
chr1	11258198	11311049	MTOR;UBIAD1
chr1	11965181	12030211	PLOD1;MFN2;MIR6729
chr1	16109337	16171069	EPHA2
chr1	16725273	17026928	RNVU1-18;RNU1-3;RNU1-4;RNU1-2;RNU1-1;MST1L;MIR3675;LOC112267871;LOC105376805;CROCC;MFAP2;ATP13A2
chr1	19200660	19266552	UBR4;EMC1-AS1;EMC1;MRTO4
chr1	20013179	20106911	PLA2G5
chr1	26417282	26486306	LIN28A;LOC101928324;DHDDS;HMGN2
chr1	36080239	36140222	TEKT2;ADPRHL2;COL8A2;TRAPPC3
chr1	40057710	40112260	CAP1;PPT1
chr1	40285489	40332813	ZMPSTE24;COL9A2
chr1	42718093	42755254	CLDN19
chr1	42910353	42973893	SLC2A1
chr1	43348398	43383074	MPL;CDC20;MIR6734;ELOVL1
chr1	43931950	43983022	ARTN;IPO13;DPH2;ATP6V0B
chr1	45485300	45528382	TESK2;CCDC163;MMACHC;PRDX1
chr1	46173683	46235305	PIK3R3;LOC110117498-PIK3R3;P3R3URF;TSPAN1;POMGNT1;LURAP1
chr1	47401285	47433052	FOXE3
chr1	58560433	58592252	TACSTD2

chr1	63307567	63340128	LINC00466;FOXD3-AS1;FOXD3;MIR6068
chr1	68413822	68464954	RPE65
chr1	93977834	94136148	ABCA4
chr1	97062743	98010000	DPYD-AS1;DPYD-AS2;DPYD;LINC01930
chr1	102861467	103123872	COL11A1
chr1	108119043	108215849	SLC25A24
chr1	108866885	108978527	GPSM2;CLCC1
chr1	109533615	109634929	GPR61;MIR197;GNAI3;GNAT2;AMPD2
chr1	110044870	110085672	STRIP1;ALX3
chr1	111102163	111155203	DRAM2
chr1	119896553	120115779	NOTCH2
chr1	145896350	145933717	ITGA10;PEX11B;GNRHR2;RBM8A
chr1	147887795	147924269	GJA8
chr1	150306479	150368233	MRPS21;PRPF3
chr1	150534369	150575937	MIR4257;ADAMTSL4;ADAMTSL4-AS1
chr1	155595205	155629951	MSTO2P;MSTO1
chr1	156132366	156192752	LMNA;SEMA4A
chr1	160261807	160301348	DCAF8;LOC100287049;PEX19
chr1	161182104	161229723	ADAMTS4;NDUFS2;FCER1G;APOA2;MIR5187;TOMM40L
chr1	161751298	161992574	DUSP12;ATF6
chr1	171620417	171667688	MYOCOS;MYOC
chr1	185719391	186205949	HMCN1
chr1	196636754	196767476	CFH
chr1	197253204	197493455	CRB1
chr1	202925826	202973572	KLHL12;ADIPOR1;CYB5R1
chr1	211461522	211507162	RD3
chr1	211643657	211690630	LINC01693;NEK2;LOC91548
chr1	212609474	212641775	ATF3;FAM71A

chr1	212843275	212914363	SPATA45;FLVCR1-DT;FLVCR1
chr1	215607891	216438448	KCTD3;LOC102723833;USH2A
chr1	220133293	220287529	IARS2;MIR664A;SNORA36B;AURKAP1;RAB3GAP2
chr1	223764893	223860954	CAPN2;TP53BP2
chr1	235432190	235519452	TBCE;B3GALNT2
chr1	235646041	235898640	GNG4;MIR1537;LYST
chr1	243241034	243515091	CEP170;MIR4677;SDCCAG8
chr1	248308630	248339568	OR2M7
chr10	1034538	1147384	IDI2-AS1;IDI1;WDR37
chr10	7688316	7764520	ITIH2
chr10	13084449	13153308	CCDC3;OPTN
chr10	13262796	13317412	PHYH
chr10	16808966	17144811	RSU1;CUBN
chr10	17213241	17252593	VIM-AS1;VIM
chr10	24833614	24967606	PRTFDC1
chr10	27095111	27258046	ANKRD26;YME1L1;MASTL;ACBD5;LRRC37A6P
chr10	27489174	27558207	RAB18
chr10	30294801	30389448	MTPAP;MIR7162;GOLGA2P6
chr10	31303495	31544814	ZEB1-AS1;LOC100505502;ZEB1
chr10	49439168	49554538	PGBD3;ERCC6
chr10	53787771	55642942	MIR548F1;LOC105378311;PCDH15;MTRNR2L5
chr10	68215595	68247113	ATOH7
chr10	69254984	69416884	HKDC1;HK1;TACR2
chr10	71381920	71830947	CDH23-AS1;CDH23;C10orf105;VSIR;MIR7152
chr10	74809927	75047624	LOC101929165;SNORD172;KAT6B
chr10	75416624	76575168	MIR606;LOC105378367;LRMDA
chr10	84179537	84274960	C10orf99;CDHR1;LRIT2;LRIT1;RGR
chr10	86741601	86947825	BMPR1A

chr10	89415299	89571641	IFIT5;SLC16A12-AS1;SLC16A12
chr10	92559105	92670395	IDE;KIF11
chr10	93576687	93681010	FFAR4;RBP4;PDE6C
chr10	95590941	95709143	ALDH18A1;TCTN3
chr10	100720396	100844944	PAX2
chr10	100992679	101047295	TWNK;LZTS2;PDZD7;SFXN3
chr10	102215186	102256512	ELOVL3;PITX3
chr10	102658731	102729397	ARL3
chr10	110883730	110934201	MIR4680;PDCD4;BBIP1
chr10	117113521	117153301	SHTN1;VAX1
chr10	122439653	122529907	PLEKHA1;ARMS2;HTRA1
chr10	124382303	124433976	OAT
chr10	127722185	127756183	FOXI2
chr11	1737752	1779573	IFITM10;CTSD
chr11	6597768	6634448	RRP8;ILK;TAF10;TPP1
chr11	8004244	8121243	CASC23;TUB-AS1;TUB
chr11	9761776	10319877	SBF2-AS1;LOC101928008;SBF2;ADM
chr11	12659421	12959737	TEAD1
chr11	14862440	14907231	PDE3B;CYP2R1
chr11	17478895	17559416	USH1C
chr11	31494755	31832961	IMMP1L;ELP4;PAX6;PAUPAR
chr11	32372775	32450564	WT1;WT1-AS
chr11	35647775	35833007	TRIM44
chr11	45894663	45933812	MAPK8IP1;C11orf94;PEX16;LARGE2
chr11	46686030	46720912	ARHGAP1;ZNF408
chr11	47199465	47254217	DDB2;ACP2
chr11	47550336	47599562	CELF1;PTPMT1;KBTBD4;NDUFS3;FAM180B;C1QTNF4
chr11	47701494	47782443	AGBL2;FNBP4

chr11	59127748	59170039	FAM111A-DT;FAM111A
chr11	61284451	61413866	VWCE;DDB1;TKFC;CYB561A3;TMEM138;TMEM216
chr11	61737636	61803518	DAGLA;MYRF-AS1;MYRF;MIR611;TMEM258;FEN1
chr11	61935063	61980515	BEST1;FTH1
chr11	62322448	62408412	ASRGL1
chr11	62596722	62630116	MTA2;EML3;ROM1;B3GAT3
chr11	65029818	65073553	SNX15;ARL2-SNX15;SAC3D1;NAALADL1
chr11	66495606	66583879	DPP3;BBS1;ZDHHC24;ACTN3;CTSF
chr11	67437406	67476752	PTPRCAP;CORO1B;GPR152;CABP4;TMEM134
chr11	68297591	68464275	LRP5
chr11	70188296	70222390	ANO1;FADD
chr11	71413193	71467868	FLJ42102;DHCR7
chr11	77051961	77230241	OMP;CAPN5;MYO7A
chr11	85632967	85671547	TMEM126B;TMEM126A;CREBZF
chr11	86930679	86970395	PRSS23;FZD4
chr11	89162875	89310759	TYR
chr11	102095447	102248424	YAP1
chr11	103094410	103494863	DYNC2H1
chr11	111893564	111938722	CRYAB;HSPB2;C11orf52;HSPB2-C11orf52
chr11	116758799	116803039	BUD13;ZPR1;APOA5
chr11	116820751	116852622	APOA4;APOC3;APOA1
chr11	117299557	117428266	BACE1;CEP164
chr11	119323942	119361705	RNF26;C1QTNF5;MFRP
chr11	121277681	121328410	SC5D
chr11	134054071	134167001	SNORD153;JAM3
chr12	1776963	1933666	ADIPOR2;LRTM2;CACNA2D4
chr12	6824954	6862393	GPR162;P3H3;GNB3;CDCA3
chr12	6927978	6961003	ATN1;RNU7-1;C12orf57;PTPN6

chr12	7173685	7233574	PEX5
chr12	7674784	7710775	GDF3
chr12	14958042	14996865	ARHGDIB;PDE6H
chr12	32664200	32760650	DNM1L;YARS2
chr12	47957967	48019554	TMEM106C;COL2A1
chr12	49003975	49075794	PRKAG1;DDN-AS1;KMT2D;RHEBL1
chr12	52774685	52811117	KRT76;KRT3
chr12	55705367	55739705	ITGA7;BLOC1S1;RDH5;BLOC1S1-RDH5;CD63
chr12	56434502	56484166	TIMELESS;MIP;SPRYD4;GLS2
chr12	57767761	57823071	METTL1;EEF1AKMT3;TSFM;AVIL
chr12	63764833	63824792	RXYLT1;RXYLT1-AS1;MIR10527
chr12	66332431	67084162	HELB;GRIP1
chr12	69570426	69616570	FRS2;MIR3913-2;MIR3913-1;CCT2;LRRC10
chr12	76329474	76363415	BBS10
chr12	85265220	85316784	ALX1
chr12	88034016	88157099	C12orf29;CEP290
chr12	89404718	89541047	GALNT4;POC1B;POC1B-GALNT4
chr12	91035491	91073024	KERA
chr12	91125484	91198217	DCN
chr12	101730499	101845959	SYCP3;GNPTAB
chr12	109558255	109613125	MMAB;MVK
chr12	110109335	110233793	IFT81
chr12	110599027	110678431	TCTN1
chr12	123218385	123273079	MPHOSPH9;C12orf65;CDK2AP1
chr12	123656110	123723399	GTF2H3;TCTN2
chr13	20123255	20176052	GJA3
chr13	26542683	26703948	WASF3
chr13	27605742	27759237	LNX2;POLR1D

chr13	31184975	31347276	B3GLCT
chr13	38672077	38902131	LINC00437;FREM2-AS1;FREM2
chr13	48217612	48285357	ITM2B
chr13	48288744	48614436	RB1-DT;LPAR6;RB1;RCBTB2;LINC00462
chr13	49516946	49600558	PHF11;SETDB2-PHF11;RCBTB1
chr13	76975660	77034143	CLN5;FBXL3
chr13	99966784	100001765	ZIC5;ZIC2;LINC00554
chr13	102830831	102890995	BIVM;ERCC5;BIVM-ERCC5
chr13	110133963	110322157	COL4A1
chr13	113652219	113752736	ATP4B;GRK1;LINC00552
chr14	21265083	21366301	HNRNPC;RPGRIP1
chr14	21506080	21552216	METTL3;SALL2
chr14	24063662	24130010	CARMIL3;CPNE6;NRL;PCK2;DCAF11
chr14	24224643	24257674	NEDD8;NEDD8-MDP1;GMPR2;TINF2
chr14	39016919	39124646	SEC23A;SEC23A-AS1
chr14	53934736	53973761	MIR5580;BMP4
chr14	56784905	56831693	OTX2
chr14	58412385	58566297	TIMM9;KIAA0586
chr14	60494146	60527850	SIX6
chr14	67661800	67831590	VTI1B;RDH11;RDH12;ZFYVE26
chr14	69839131	70047366	SNORD169;SMOC1
chr14	73260107	73473617	PAPLN;NUMB;LOC101928143
chr14	74224449	74277738	VSX2
chr14	74483183	74627378	NPC2;ISCA2;LTBP2
chr14	75618625	75970079	FLVCR2;ERG28;TTLL5
chr14	77259956	77335883	MIR1260A;NGB;POMT2;GSTZ1
chr14	88369924	88485350	SPATA7
chr14	88809153	88896078	TTC8

chr14	91854411	91962987	TC2N;FBLN5
chr14	102907663	102948596	TRAF3;AMN
chr14	104909713	104952761	PLD4
chr15	27739875	28114315	OCA2
chr15	30986061	31176273	MTMR10;MIR211;TRPM1
chr15	43354221	43424771	ADAL;ZSCAN29;TUBGCP4
chr15	48105990	48157672	SLC24A5
chr15	48393313	48660721	FBN1
chr15	65077760	65130200	KBTBD13;UBAP1L
chr15	65596366	65675995	INTS14;SLC24A1
chr15	68191992	68272211	CALML4;CLN6
chr15	72671179	72753475	HIGD2B;BBS4
chr15	74164466	74227267	ISLR;STRA6
chr15	76332904	76920444	ISL2;MIR3713;SCAPER
chr15	78089606	78186945	SH2D7;CIB2;IDH3A
chr15	79194788	79224871	MIR184
chr15	82644281	82725112	CPEB1;AP3B2;CPEB1-AS1;LOC338963
chr15	86064871	87064169	LINC01584;AGBL1-AS1;LOC105370954;LOC102724452;AGBL1
chr15	89194869	89349861	ABHD2;RLBP1;FANCI;MIR6766;POLG
chr15	89593789	89678086	TICRR;KIF7;PLIN1
chr15	99956437	100357005	ADAMTS17;SPATA41
chr15	100862714	100931626	LOC105369201;ALDH1A3;LOC101927751
chr16	1336931	1380737	BAIAP3;TSR3;GNPTG;UNKL
chr16	1495427	1627072	TELO2;TMEM204;LOC105371046;IFT140
chr16	1969193	2002749	NOXO1;TBL3;GFER;SYNGR3
chr16	2260881	2355746	RNPS1;MIR3677;MIR940;LOC106660606;MIR4717;ABCA3
chr16	3485976	3554048	NAA60;C16orf90;CLUAP1
chr16	13905138	13967348	ERCC4

chr16	16134565	16238522	ABCC1;ABCC6
chr16	28459111	28510575	CLN3;APOBR;IL27
chr16	30009427	30067978	DOC2A;C16orf92;TLCD3B;LOC112694756
chr16	49472524	49872919	ADAM3B;ZNF423
chr16	51120982	51167334	SALL1
chr16	53583153	53718938	RPGRIP1L
chr16	54915865	54949485	CRNDE;IRX5
chr16	55309203	55345756	IRX6
chr16	56450640	56597667	NUDT21;OGFOD1;BBS2;MT4;MT3
chr16	57230259	57268635	RSPRY1;ARL2BP
chr16	57867340	57986128	CNGB1
chr16	67149681	67184945	B3GNT9;TRADD;FBXL8;HSF4;NOL3;KIAA0895L
chr16	67924750	67959131	PSKH1;CTRL;PSMB10;LCAT
chr16	68621189	68742468	CDH3
chr16	72078613	72127912	TXNL4B;DHX38
chr16	75457052	75510445	TMEM170A;CHST6
chr16	75521741	75571289	CHST5;TMEM231
chr16	77232813	77450034	ADAMTS18
chr16	79570843	79615737	MAF
chr16	83994667	84057795	NECAB2;SLC38A8
chr16	88367959	88455757	ZNF469
chr16	89475719	89572766	ANKRD11;SPG7;SNORD68;RPL13
chr16	89711683	89831977	VPS9D1-AS1;VPS9D1;ZNF276;FANCA
chr17	1635629	1699867	SCARF1;RILP;PRPF8
chr17	4917277	4950023	GP1BA;SLC25A11;RNF167;PFN1
chr17	6378693	6571555	AIPL1;PIMREG;PITPNM3
chr17	7987615	8035342	GUCY2D
chr17	8209815	8263056	AURKB;LINC00324;CTC1

chr17	16202191	16366797	NCOR1;MIR1288;PIGL;CENPV
chr17	28531708	28567631	FOXN1;UNC119
chr17	29231859	29269494	CRYBA1
chr17	32912910	32960106	TMEM98
chr17	34965512	35024743	LIG3
chr17	35559795	35593863	SNORD7;SNHG30;PEX12
chr17	40294180	40372643	CDC6;RARA-AS1;RARA;GJD3
chr17	40846303	40882223	KRT12
chr17	44330246	44368106	GRN;FAM171A2
chr17	44834948	44914445	HIGD1B;EFTUD2;CCDC103;FAM187A
chr17	46747506	46848154	NSF;WNT3
chr17	58190441	58234605	EPX;MKS1
chr17	60134942	60185899	CA4
chr17	65085812	65242703	LOC100507002;RGS9
chr17	68244182	68437731	AMZ2;SLC16A6;ARSG;MIR635
chr17	74901083	74938256	USH1G;OTOP2
chr17	75736594	75780236	ITGB4;GALK1;H3F3B
chr17	76512586	76568578	CYGB;PRCD;SNORD1C;SNORD1B;SNORD1A;SNHG16
chr17	81494413	81552130	ACTG1;FSCN2
chr17	81635459	81678112	NPLOC4;TSPAN10;PDE6G;OXLD1;CCDC137
chr18	2640726	2820017	CBX3P2;SMCHD1
chr18	6926742	7132797	LINC00668;LOC101927188;LAMA1
chr18	12313944	12392227	TUBB6;AFG3L2
chr18	34961928	35158470	MAPRE2
chr18	45785581	45982329	SIGLEC15;EPG5
chr18	51013394	51100045	SMAD4
chr18	59252035	59289086	RAX
chr18	68658688	68730108	TMX3

chr18	79664803	79769503	LOC284241;CTDP1
chr19	1476166	1512927	C19orf25;PCSK4;REEP6;ADAMTSL5
chr19	3754089	3787228	APBA3;MRPL54;RAX2;MATK
chr19	5850826	5885540	FUT3;LOC101928844;FUT5
chr19	6662704	6745562	TNFSF14;C3;MIR6791;GPR108
chr19	7333937	7487485	ARHGEF18
chr19	7519004	7576764	ZNF358;MCOLN1;PNPLA6
chr19	8565240	8625735	MYO1F;ADAMTS10
chr19	9838718	9951515	PIN1;OLFM2
chr19	11184295	11277524	KANK2;LOC105372273;ANGPTL8;DOCK6
chr19	12631511	12681742	ZNF791;MAN2B1;WDR83OS;WDR83;DHPS
chr19	13191442	13648025	CACNA1A
chr19	13916187	13968392	CC2D1A;PODNL1;DCAF15
chr19	16877951	17041815	SIN3B;F2RL3;CPAMD8
chr19	29683937	29730789	C19orf12
chr19	32660848	32693300	ANKRD27;RGS9BP
chr19	38229035	38307615	DPF1;PPP1R14A;SPINT2;C19orf33
chr19	43712983	43769962	IRGC;SMG9
chr19	45334837	45385918	KLC3;ERCC2
chr19	45392334	45493828	PPP1R13L;CD3EAP;ERCC1;MIR6088;FOSB;RTN2
chr19	45512767	45617212	VASP;OPA3;GPR4
chr19	46731046	46791988	STRN4;FKRP;SLC1A5
chr19	47804779	47858330	CRX
chr19	48950309	48981879	BAX;FTL
chr19	49046066	49080076	CGB8;CGB7;NTF4;KCNA7
chr19	49576182	49641439	NOSIP;PRRG2;PRR12;RRAS
chr19	49803282	49855383	AP2A1;FUZ;MIR6800;MED25;PTOV1-AS1;MIR4749
chr19	51364909	51402974	ETFB;CLDND2;NKG7;LIM2;C19orf84

chr19	54100410	54146719	OSCAR;NDUFA3;TFPT;PRPF31
chr2	1616887	1759852	PXDN
chr2	15151914	15576334	NBAS
chr2	27027364	27085622	TMEM214;AGBL5-AS1;AGBL5;OST4
chr2	27362235	27395790	EIF2B4;SNX17;ZNF513;FTH1P3
chr2	27429377	27504805	NRBP1;KRTCAP3;IFT172;FNDC4
chr2	29045976	29089523	TOGARAM2;PCARE
chr2	36340778	36566135	CRIM1-DT;CRIM1
chr2	38051973	38124902	RMDN2;CYP1B1
chr2	44926702	44961071	LINC01833;SIX3-AS1;SIX3
chr2	55850967	55939139	EFEMP1
chr2	58144243	58256410	VRK2;FANCL
chr2	61002225	61066990	PUS10;PEX13
chr2	61809848	61869143	FAM161A
chr2	63104559	63842843	DBIL5P2;WDPCP;MDH1
chr2	73370758	73640166	ALMS1-IT1;ALMS1
chr2	73911826	73973961	ACTG2;DGUOK
chr2	96259338	96336271	TMEM127;CIAO1;SNRNP200;ITPRIPL1
chr2	96745902	96826874	MIR3127;CNNM4;CNNM3-DT
chr2	98331188	98413601	CNGA3
chr2	110107311	110220066	MALL;NPHP1;MTLN
chr2	111883607	112044561	ANAPC1;MERTK
chr2	120720623	121007653	GLI2
chr2	127242290	127309166	ERCC3
chr2	135037289	135191667	SNORA40B;RAB3GAP1
chr2	144349364	144536057	ZEB2;ZEB2-AS1;LOC105373656
chr2	165842475	165968851	LOC100506124;TTC21B-AS1;TTC21B
chr2	167278001	167889045	B3GALT1

chr2	169112109	169377534	LRP2
chr2	169464480	169521655	BBS5
chr2	177377746	177582024	LOC100130691;AGPS;TTC30B
chr2	181520041	181695827	ITGA4;CERKL;NEUROD1
chr2	190865821	190980552	GLS
chr2	201605184	201658570	C2CD6;TMEM237
chr2	206099817	206174509	GCSHP3;NDUFS1;SNORD51;SNORA41;EEF1B2
chr2	207747598	207784906	MIR4775;CCNYL1;FZD5
chr2	208106607	208161158	CRYGD;CRYGC;CRYGB;LOC100507443
chr2	208251255	208373746	IDH1;IDH1-AS1;PIKFYVE
chr2	218766749	218830293	CYP27A1;MIR9500
chr2	219194772	219233994	ZFAND2B;ABCB6;ATG9A
chr2	222184887	222313998	PAX3;CCDC140
chr2	227310151	227372836	COL4A3;LOC654841;MFF
chr2	231717433	231801272	PDE6D
chr2	232505388	232540716	PRSS56;CHRND
chr2	232750802	232791565	KCNJ13
chr2	233292816	233362055	ATG16L1;SAG
chr2	234035679	234092134	SPP2
chr2	238305441	238415897	TRAF3IP1
chr20	408596	477566	RBCK1;TBC1D20
chr20	2643395	2679219	MIR1292;SNORD110;SNORA51;SNORD86;SNORD56;SNORD57;NOP56;IDH3B
chr20	3193868	3254559	DDRGK1;ITPA;SLC4A11
chr20	3873839	3944887	MAVS;PANK2;MIR103B2;MIR103A2
chr20	10386009	10449222	MKKS
chr20	10622684	10688999	SLX4IP;MIR6870;JAG1
chr20	17478905	17584220	PCSK2;BFSP1
chr20	17941979	18074188	SNORD17;SNX5;MGME1;OVOL2

chr20	21110983	21261622	KIZ-AS1;KIZ
chr20	25055885	25097141	ACSS1;VSX1
chr20	25279742	25405835	PYGB;ABHD12
chr20	32262651	32350011	KIF3B
chr20	33796348	33869366	ZNF341-AS1;CHMP4B
chr20	35440164	35534280	MIR1289-1;GDF5;CEP250;C20orf173
chr20	51767331	51817521	ATP9A;SALL4
chr20	57153753	57281641	BMP7-AS1;BMP7
chr20	63966132	64048100	ZNF512B;SAMD10;PRPF6;C20orf204
chr21	41724369	41782089	MIR6814;RIPK4
chr21	43038191	43091943	CBS;CBSL
chr21	43154008	43187805	CRYAA;CRYAA2
chr21	44270838	44313648	AIRE
chr21	44313944	44354402	PFKL;CFAP410
chr21	45390165	45528720	COL18A1-AS2;COL18A1-AS1;MIR6815;COL18A1
chr22	18062923	18120396	PEX26
chr22	25184858	25246870	KIAA1671;CRYBB3;CRYBB2
chr22	26584278	26645669	TPST2;CRYBB1;CRYBA4
chr22	29465218	29506390	NEFH
chr22	29588556	29713598	NF2
chr22	32786705	32878041	TIMP3
chr22	36266280	36403010	APOL1;MIR6819;MYH9
chr22	36743202	36791256	LOC105373021;IFT27
chr22	41432830	41544273	TOB2;PHF5A;ACO2;POLR3H
chr22	50202689	50260023	SELENOO;TUBGCP6;HDAC10
chr3	3111933	3165879	TRNT1
chr3	4478345	4862506	ITPR1-DT;EGOT;ITPR1
chr3	10011370	10116932	CIDECP1;FANCD2;FANCD2OS

chr3	14130147	14193621	TMEM43;XPC
chr3	14387576	14504349	SLC6A6
chr3	24672887	25612932	RARB-AS1;RARB
chr3	41179741	41275096	CTNNB1
chr3	43064229	43121085	POMGNT2
chr3	45808316	46010824	LZTFL1;CCR9;CXCR6;FYCO1
chr3	48450811	48482645	ATRIP;TREX1;ATRIP-TREX1;SHISA5
chr3	49106114	49148118	USP19;LAMB2
chr3	49453703	49550618	DAG1
chr3	50176610	50212696	SEMA3F;GNAT1
chr3	50596520	50664291	CISH;MAPKAPK3
chr3	63848155	64018462	C3orf49;THOC7-AS1;THOC7;SCAANT1;ATXN7;PSMD6-AS2
chr3	69724456	69983336	MITF
chr3	93965139	94070678	PROS1;STX19;ARL13B;DHFR2
chr3	97749521	97816229	EPHA6;ARL6
chr3	101207546	101335575	IMPG2
chr3	121754761	121850079	IQCB1
chr3	129513639	129550344	IFT122;RHO
chr3	132665609	132737432	UBA5;NPHP3;NPHP3-ACAD11
chr3	133385056	133490222	TMEM108;BFSP2-AS1;BFSP2
chr3	138929224	138962137	LINC01391;FOXL2;FOXL2NB
chr3	150911163	150987727	CLRN1
chr3	155806024	155869456	C3orf33;SLC33A1
chr3	170444548	170601075	SLC7A14-AS1;SLC7A14
chr3	180968697	181004774	FXR1;DNAJC19
chr3	181696925	181729436	SOX2
chr3	184227301	184264548	MIR1224;VWA5B2;ALG3;EEF1AKMT4;CAMK2N2
chr3	186523441	186561702	CRYGS

chr3	189941728	190137437	P3H2
chr3	193578144	193712811	OPA1-AS1;OPA1
chr3	196199222	196302957	ZDHHC19;SLC51A;PCYT1A
chr3	196691277	196727250	CEP19
chr4	610573	685782	PDE6B;ATP5ME;MYL5
chr4	6254849	6318265	WFS1
chr4	8831076	8886839	HMX1
chr4	13346354	13499365	RAB28
chr4	15454865	15616552	CC2D2A
chr4	15948076	16099378	FGFBP2;PROM1
chr4	22330071	22531066	LOC100505912;MIR12115;ADGRA3
chr4	36266616	36362511	LOC439933;DTHD1
chr4	39167504	39300810	WDR19
chr4	47920977	48031681	CNGA1;LOC101927157
chr4	55331213	55388100	SRD5A3;SRD5A3-AS1
chr4	71047667	71587087	SLC4A4
chr4	78042323	78559269	SNORD161;FRAS1
chr4	99549081	99638997	TRMT10A;MTTP
chr4	102853974	102907807	UBE2D3;CISD2
chr4	109833107	109887315	RRH;LRIT3
chr4	110602423	110657123	PANCR;PITX2
chr4	112270509	112457621	AP1AR;TIFA;ALPK1
chr4	120669919	120937870	PRDM5
chr4	121809329	121885487	EXOSC9;CCNA2;BBS7
chr4	122137331	122379167	KIAA1109
chr4	122717702	122759942	CETN4P;BBS12
chr4	127865893	127981034	PLK4;MFSD8
chr4	128849921	129108600	JADE1;SCLT1

chr4	150567151	150599693	LOC729558;MAB21L2
chr4	154611945	154768120	FGG;LRAT
chr4	182128987	182818024	TENM3-AS1;MIR1305;TENM3
chr4	186176567	186228463	FLJ38576;CYP4V2
chr4	186572794	186741722	FAT1
chr5	203303	272082	CCDC127;SDHA;HRAT5
chr5	33929623	34023104	RXFP3;SLC45A2;AMACR
chr5	37091228	37264376	CPLANE1;LOC105374727
chr5	60851454	60960073	ERCC8
chr5	64751368	65117412	SREK1IP1;CWC27
chr5	68200756	68316821	PIK3R1
chr5	75659124	75732448	ANKDD1B;POC5;LOC441087
chr5	83456618	83597303	VCAN-AS1;VCAN
chr5	90514344	91179437	POLR3G;LYSMD3;ADGRV1
chr5	93568222	93609611	NR2F1-AS1;NR2F1
chr5	110723136	110780161	TMEM232;SLC25A46
chr5	111077321	111145502	TSLP;WDR36
chr5	136013988	136078818	TGFBI
chr5	138595967	139308557	LOC105379194;LRRTM2;CTNNA1;SIL1;SNORA74D;SNORA74A;SNHG4
chr5	140658035	140706537	IK;WDR55;DND1;HARS;HARS2;ZMAT2
chr5	149842953	149959793	PPARGC1B;LOC644762;PDE6A
chr5	150342629	150415308	TCOF1;CD74
chr5	172310000	172469525	SH3PXD2B
chr5	178962587	179011206	ZNF454;GRM6
chr6	1594915	1628897	FOXCUT;FOXC1
chr6	10378186	10434659	TFAP2A-AS2;TFAP2A;TFAP2A-AS1
chr6	10477223	10644368	GCNT2
chr6	10747723	10853553	TMEM14B;MAK

chr6	30702435	30740538	MDC1-AS1;MDC1;TUBB
chr6	31882785	31967084	EHMT2;ZBTB12;C2;C2-AS1;CFB;MIR1236;NELFE
chr6	33147681	33207499	HCG24;COL11A2;RXRB;SLC39A7;HSD17B8
chr6	35437338	35482104	FANCE;MIR7111;RPL10A
chr6	35482874	35527896	TEAD3;TULP1
chr6	42140406	42209956	C6orf132;LOC114841037;GUCA1A;GUCA1B
chr6	42681598	42737597	UBR2;PRPH2;ATP6V0CP3
chr6	42948865	42994181	CNPY3-GNMT;GNMT;PEX6
chr6	43494702	43635523	TJAP1;LRRC73;YIPF3;POLR1C;XPO5;POLH;GTPBP2
chr6	63704980	65722226	PHF3;SCAT8;LOC441155;EYS
chr6	70201040	70318084	COL19A1;COL9A1
chr6	71871550	72418145	RIMS1
chr6	75906114	76087678	MYO6;IMPG1
chr6	79469991	79552458	LCA5
chr6	79899814	79962553	ELOVL4
chr6	99417325	99536728	PNISR;LOC101927365;USP45;TSTD3
chr6	99591774	99630578	PRDM13
chr6	106555771	106644498	CRYBG1;RTN4IP1
chr6	108196222	108276246	SNX3
chr6	121064494	121349745	TBC1D32
chr6	121420595	121464727	GJA1
chr6	135268407	135513434	AHI1
chr6	136807564	136928934	PEX7;SLC35D3
chr6	143435805	143505616	ADAT2;PEX3
chr6	158153350	158214344	SERAC1;GTF2H5
chr7	5511409	5578902	FBXL18;LOC221946;ACTB
chr7	6779134	6841770	RSPH10B2;RSPH10B;CCZ1B
chr7	16072525	16517504	CRPPA-AS1;CRPPA;SOSTDC1

chr7	16901359	17361152	AHR
chr7	22874371	23029130	FAM126A
chr7	23090758	23192914	KLHL7-DT;KLHL7
chr7	33079797	33892180	RP9;BBS9
chr7	40111027	40149622	MPLKIP
chr7	66613881	66664067	KCTD7
chr7	76594986	76642279	POMZP3;LOC100133091
chr7	83348238	83664139	SEMA3E
chr7	92069987	92149803	AKAP9;CYP51A1
chr7	92472020	92543520	ERVW-1;PEX1;RBM48
chr7	99377048	99423597	ARPC1B;PDAP1;MIR12119;BUD31
chr7	120772320	120873402	TSPAN12
chr7	128377277	128425252	LOC107986845;IMPDH1
chr7	128757485	128790794	CALU;OPN1SW
chr7	129173633	129228545	SMO
chr7	130378771	130457433	CPA1;CEP41
chr7	138816381	138996389	KIAA1549
chr7	140278693	140419433	SLC37A3
chr7	141536278	141670244	AGK
chr7	141723334	141802922	WEE2;WEE2-AS1;SSBP1;TAS2R3;TAS2R4;TAS2R5
chr7	151160698	151202832	GBX1;ASB10
chr7	155784980	155827463	SHH
chr8	1740778	1816711	LOC101927752;CLN8;MIR3674
chr8	10591349	10727187	RP1L1;MIR4286;C8orf74
chr8	27756949	27827640	CCDC25;ESCO2
chr8	31018788	31191138	PURG;WRN
chr8	38981754	39120445	HTRA4;TM2D2;SNORD38D;ADAM9
chr8	43125464	43217855	HGSNAT

chr8	54494422	54886720	RP1
chr8	60663740	60883028	CHD7
chr8	63033553	63101053	GGH;TTPA
chr8	67047417	67211778	COPS5;CSPP1
chr8	71182433	71607025	EYA1
chr8	76965258	77016044	MIR3149;PEX2
chr8	86538977	86758675	CPNE3;CNGB3
chr8	93739844	93834234	RBM12B-AS1;RBM12B;TMEM67
chr8	95229913	95284201	C8orf37
chr8	96127333	96175806	GDF6
chr8	98998266	99892580	VPS13B;MIR599;MIR875;COX6C
chr8	103485610	104269430	LOC105375690;RIMS2
chr8	143801344	143844352	MIR937;SCRIB;PUF60;MIR6845;NRBP2
chr8	144318957	144376286	DGAT1;SCRT1;TMEM249;FBXL6;SLC52A2;LOC101928902
chr9	2702510	2745037	KCNV2
chr9	12670439	12725285	TYRP1
chr9	14722152	14925995	CER1;FREM1
chr9	26932039	27081134	PLAA;IFT74-AS1;LRRC19;IFT74
chr9	32440302	32567586	ACO1;DDX58;TOPORS;SMIM27
chr9	34623133	34666035	ARID3C;SIGMAR1;GALT;IL11RA;CCL27;LOC730098
chr9	69020751	69094076	FXN
chr9	70794975	70825084	MIR204
chr9	78221062	78294690	CEP78
chr9	91198815	91376918	AUH
chr9	95427980	95532057	LOC100507346;PTCH1
chr9	97397096	97511125	TDRD7
chr9	97659909	97712340	NCBP1;XPA
chr9	100084243	100317175	ERP44;INVS

chr9	105543122	105668820	FSD1L;FKTN;TAL2
chr9	113260642	113309009	SLC31A1;CDC26;PRPF4;RNF183
chr9	114387080	114520473	AKNA;WHRN
chr9	116672305	116716300	ASTN2;TRIM32
chr9	121192794	121347843	RAB14;GSN-AS1;GSN
chr9	126598928	126716032	LMX1B
chr9	130678721	130722288	PRDM12;EXOSC2
chr9	131487789	131538806	SNORD62A;SNORD62B;PRRC2B;POMT1;UCK1
chr9	136413619	136454845	PMPCA;INPP5E
chr9	137226287	137258707	CYSRT1;RNF224;SLC34A3;TUBB4B;FAM166A;STPG3;STPG3-AS1
chrM	0	16569	RNR1;RNR2;MIR12136
chrX	9710346	9801297	TBL1X;GPR143
chrX	11096301	11138086	HCCS
chrX	13719743	13792955	TRAPPC2;OFD1
chrX	17360200	17750994	MIR4768;NHS-AS1;LOC101928389;NHS
chrX	18624688	18687108	CDKL5;RS1
chrX	31082677	33354609	DMD;MIR3915;MIR548F5
chrX	38254163	38342544	RPGR
chrX	40034815	40192329	BCOR
chrX	41432343	41490710	NYX
chrX	43933776	43988395	NDP-AS1;NDP
chrX	44858188	45127779	KDM6A
chrX	46822043	46897358	RP2
chrX	47127071	47160466	NDUFB11
chrX	48493959	48535814	LOC101927635;PORCN;EBP
chrX	48786377	48839982	GATA1;HDAC6;ERAS;PCSK1N
chrX	48875197	48918402	TIMM17B;PQBP1;SLC35A2
chrX	49190063	49248371	SYP;SYP-AS1;CACNA1F

chrX	70118447	70181324	IGBP1
chrX	77884440	78144295	MAGT1;COX7B;PGAM4;ATP7A;PGK1;TAF9B
chrX	79999753	80046774	TBX22
chrX	85846180	86062561	MIR361;CHM
chrX	101330661	101363742	TIMM8A
chrX	107613428	107666993	PRPS1
chrX	110658856	110810819	CHRDL1
chrX	129524849	129607561	OCRL
chrX	132061990	132143020	STK26;FRMD7
chrX	150965509	151005771	HMGB3
chrX	153914225	153950080	ARHGAP4;NAA10;RENBP
chrX	154129243	154174032	OPN1LW
